# Lack of cellular prion protein causes Amyloid β accumulation, increased extracellular vesicle abundance, and changes to exosome biogenesis proteins

**DOI:** 10.1007/s11010-024-05059-0

**Published:** 2024-07-06

**Authors:** Lovisa Johansson, Juan F. Reyes, Tahir Ali, Hermann Schätzl, Sabine Gilch, Martin Hallbeck

**Affiliations:** 1 Department of Biomedical and Clinical Sciences and Department of Clinical Pathology, Linköping University, Linköping, Sweden; 2 Calgary Prion Research Unit, Faculty of Veterinary Medicine, University of Calgary, Calgary, AB Canada; 3 Hotchkiss Brain Institute, University of Calgary, Calgary, AB Canada

**Keywords:** Alzheimer’s disease, Amyloid β, Extracellular vesicles, Exosome, Prion, ESCRT

## Abstract

Alzheimer's disease (AD) progression is closely linked to the propagation of pathological Amyloid β (Aβ), a process increasingly understood to involve extracellular vesicles (EVs), namely exosomes. The specifics of Aβ packaging into exosomes remain elusive, although evidence suggests an ESCRT (Endosomal Sorting Complex Required for Transport)-independent origin to be responsible in spreading of AD pathogenesis. Intriguingly, PrP^C^, known to influence exosome abundance and bind oligomeric Aβ (oAβ), can be released in exosomes via both ESCRT-dependent and ESCRT-independent pathways, raising questions about its role in oAβ trafficking. Thus, we quantified Aβ levels within EVs, cell medium, and intracellularly, alongside exosome biogenesis-related proteins, following deletion or overexpression of PrP^C^. The same parameters were also evaluated in the presence of specific exosome inhibitors, namely Manumycin A and GW4869. Our results revealed that deletion of PrP^C^ increases intracellular Aβ accumulation and amplifies EV abundance, alongside significant changes in cellular levels of exosome biogenesis-related proteins Vps25, Chmp2a, and Rab31. In contrast, cellular expression of PrP^C^ did not alter exosomal Aβ levels. This highlights PrP^C^’s influence on exosome biogenesis, albeit not in direct Aβ packaging. Additionally, our data confirm the ESCRT-independent exosome release of Aβ and we show a direct reduction in Chmp2a levels upon oAβ challenge. Furthermore, inhibition of opposite exosome biogenesis pathway resulted in opposite cellular PrP^C^ levels. In conclusion, our findings highlight the intricate relationship between PrP^C^, exosome biogenesis, and Aβ release. Specifically, they underscore PrP^C^’s critical role in modulating exosome-associated proteins, EV abundance, and cellular Aβ levels, thereby reinforcing its involvement in AD pathogenesis.

**Graphical abstract:**

There are two main exosome biogenesis pathways: ESCRT dependent and ESCRT independent. In this study, we explored the effect of the cellular prion protein (PrP^C^) on the release of Amyloid β via exosomes. Our findings demonstrate that Amyloid β mainly is released via an ESCRT-independent pathway, independent of PrP^C^. However, lack of PrP^C^ resulted in upregulation of the ESCRT-dependent proteins Tsg101 and VPS25, a decrease in Chmp2a, and an overall increase in extracellular vesicles. Lack of PrP^C^ also caused an accumulation of cellular, but not exosomal, Amyloid β.

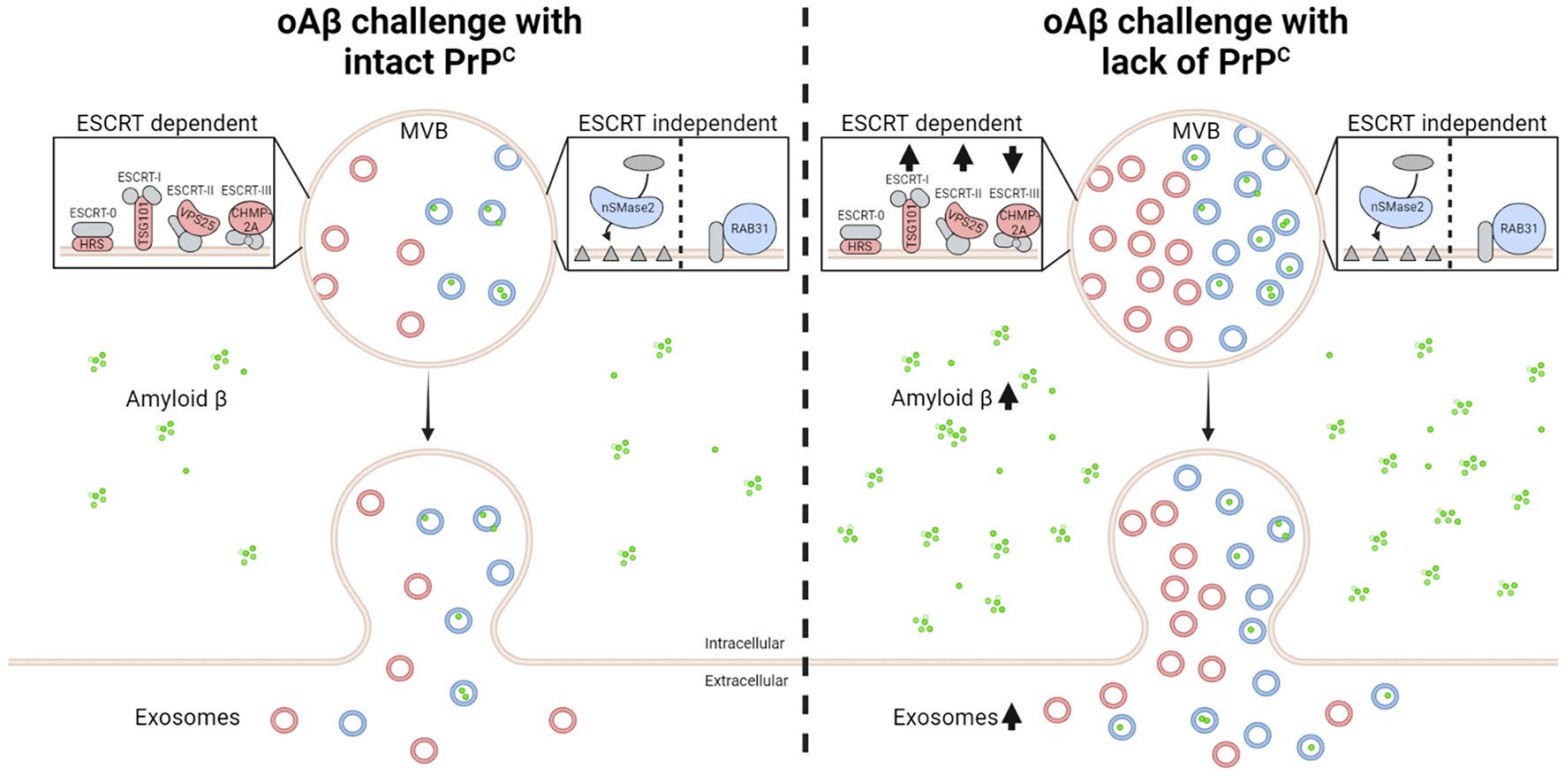

**Supplementary Information:**

The online version contains supplementary material available at 10.1007/s11010-024-05059-0.

## Introduction

Small extracellular vesicles (EVs), including exosomes, are important for cell-to-cell communication and play a significant role in the progression of multiple neurodegenerative diseases including Alzheimer’s disease (AD) [1]. These vesicles are involved in the transport of pathological protein aggregates of both Amyloid β (Aβ) and tau [2–4], which have been linked to increased neuronal loss and accelerated disease progression [5–7]. The growing interest in exosomes stems from their role in carrying these disease-related proteins, suggesting that understanding exosome-mediated transport could open new avenues for therapeutic intervention in AD and other neurodegenerative diseases [2–4].

The process by which Aβ and tau protein assemblies are selectively loaded into exosomes is still not fully understood. Since exosomes are derived from multivesicular bodies (MVBs) within the endocytic system [8], any protein in this system could end up in an exosome. However, evidence points toward cargo-loading being a highly controlled mechanism since proteins can be targeted for exosome inclusion by ubiquitination and as an alternative way of waste disposal [9]. Furthermore, the exosome composition could also impact cell-to-cell transport since exosomes have specific target proteins and can home to specific organs [10, 11]. As the spread of Aβ between neurons is linked to disease progression [1, 12], the incorporation of disease-related proteins into specific exosomes is an important mechanism to investigate. Further complicating matters, exosomes are formed by two distinct biogenesis pathways: the Endosomal Sorting Complex Required for Transport (ESCRT)-dependent and the ESCRT-independent pathways [8]. These mechanisms are believed to serve unique functions but may also compensate for each other under varying cellular conditions [13, 14], including altered expression of exosome biogenesis-related genes in AD brains [15]. Although the specifics of Aβ loading into exosomes remain elusive, current evidence suggests the involvement of the ESCRT-independent exosome pathway in AD progression [16, 17]. Indeed, reduced pathology in multiple animal models of AD has been reported when the ESCRT-independent pathway is inhibited [16, 18].

Prion disease research has revealed that both ESCRT-dependent and ESCRT-independent pathways are crucial for the release of cellular prion protein (PrP^C^) and its disease-related scrapie form (PrP^Sc^) [19–21]. Interestingly, formation of the misfolded PrP^Sc^ and its incorporation into exosomes is dependent on which exosome biogenesis-related protein is present [19], suggesting that distinct proteins within one pathway could contribute to variations in exosome cargo. Moreover, the importance of PrP^C^ in exosome release is notable as knockout of PrP^C^ was reported to reduce exosome abundance while overexpressing PrP^C^ had the opposite effect [22]. In AD however, brain-derived EVs have elevated PrP^C^ levels [23] but vesicle concentration remains the same [24], questioning PrP^C^’s relevance to EV abundance in the disease. Instead, the binding of Aβ directly to PrP^C^, including on exosomes [25, 26], could be key to understanding AD progression and pathogenesis. In fact, inhibiting the binding between PrP^C^ and oligomeric Aβ (oAβ) resulted in improved cognitive functions and reduced oAβ and plaque levels in the 5XFAD mouse model of AD [27].

In this study, we elucidated the role of PrP^C^ in the incorporation of oAβ into exosomes and its impact on the ESCRT-dependent and ESCRT-independent exosome biogenesis pathways. Thus, we employed a neuroblastoma cell model, N2a, to examine the effects of PrP^C^ on various exosome biogenesis-related proteins under physiological conditions and after oAβ challenge. Our findings demonstrate that lack of PrP^C^ resulted in differential expression of exosome biogenesis-related protein levels, an unexpected increase in EV abundance, and intracellular Aβ accumulation. Surprisingly, stimulation of N2a cells with oAβ resulted in additive effects on some exosome biogenesis-related proteins but had no downstream effects on EV abundance. Furthermore, exosomal oAβ release was shown to preferentially involve the proposed ESCRT-independent pathway. Collectively, our results shed light on the interdependent relationship between PrP^C^, exosome biogenesis, and oAβ propagation, providing insights into potential therapeutic targets to halt the progression of AD.

## Materials and methods

### Cell culturing and transfection of N2a cells

All N2a cells were cultured in DMEM-Glutamax (Gibco) media supplemented with 10% Fetal Bovine Serum (FBS, PAA Laboratories), 50 U/mL Penicillin, and 50 U/mL Streptomycin (Gibco). Cell proliferation rate and viability was measured using a TC 20™ automated cell counter (Bio-Rad) and staining of the cells using Trypan blue (Bio-Rad). To explore the effects of PrP^C^, *PRNP* was knocked out of an N2a cell using CRISPR/Cas9 (PrP KO cells) which was generously provided by Gerold Schmitt-Ulms (Toronto University) [28]. To restore PrP^C^ expression, PrP KO cells were transiently transfected using Lipofectamine LTX Reagent with PLUS Reagent (Invitrogen), along with 2 µg plasmid vector (pcDNA3.1) expressing mouse 3F4-PrP. The PrP KO cells were seeded at a density of 500,000 cells per 6 cm culture dish, and transfected 24 h later, following manufacturer’s instructions. Cell collection was carried out 3 days post-transfection and these overexpressing cells are hereon called PrP KO^OE^. A stable PrP^C^ expression cell line, hereon called N2a-PRNP, with stable transfection of murine PrP^C^ was achieved as previously described [29]. These cells were utilized for oAβ challenge and inhibitor experiments.

### Generation of oligomeric Amyloid β (oAβ)

Since PrP^C^ preferentially bind to oligomers and oAβ is the most disease-related form [30], we generated these aggregates and collected based on size. Aβ1-42 peptide (Innovagen) was oligomerized as previously described [31]. In brief, recombinant Aβ1-42 was dissolved in 1,1,1,3,3,3-hexafluoro-2-propanol (HFIP, Sigma Aldrich), lyophilized using a Savant Speed Vac Plus freeze drier (Thermo Fisher), and then resuspended in dimethyl sulfoxide. The peptides were further diluted in *N*-2-hydroxyethylpiperazine-*N*-2-ethane sulfonic acid (HEPES) buffer (20 mM, pH 7.4) to a final 100 µM concentration. The preparation was vortexed, sonicated in a water bath for 2 min, and subsequently incubated for 24 h at 4 °C. The suspension was then lyophilized and resuspended in an NH_4_HCO_3_ (50 mM, pH 8.5) buffer. The oligomeric Aβ1-42 (oAβ) was isolated using a Superdex 75 10/300 GL column coupled to a liquid chromatography system (ÄKTA pure, GE Healthcare) at 4 °C with a flow rate of 0.5 mL/min. By collecting the void volume, oAβ above 70 kDa in size was isolated. The oAβ was lyophilized overnight and then resuspended in PBS (Gibco). The concentration was quantified spectrophotometrically at 215 nm using a Nanodrop One (Thermo Scientific), with the Aβ1-42 molar absorption coefficient (ε215nm = 75,887 M^−1^ cm^−1^).

### oAβ challenge and inhibition of exosome biogenesis pathways

Cells were seeded either in 12-well plates (250 000 cells/well) for immunoblotting and RT-PCR analyses or in T75 flasks (2 million cells/flask) for immunoblotting analyses and EV isolation. The cells were cultured in DMEM-Glutamax (Gibco), supplemented with 50 U/mL Penicillin, 50 U/mL Streptomycin (Gibco), and either 10% FBS (PAA Laboratories) or 10% EV-depleted FBS (Gibco). For stimulation, cells were exposed to 2.5 µM oAβ, with or without EV inhibitors (20 µM GW4869 or 0.25 µM Manumycin A) dissolved in equal amounts of dimethyl sulfoxide (DMSO), for 6 h. At this point, medium and cells were either isolated or washed with PBS followed by an additional 48 h incubation in 25 mL cell medium for flasks and 2.5 mL for 12-well plates, with or without EV inhibitors. The control medium contained the same amount of DMSO as the EV-inhibitor medium (0.58%). After 48 h, cells were collected for immunoblotting purposes and frozen until further use. Cell medium was collected for EV isolation and centrifuged at 2000xg for 10 min using a TX-400 swinging bucket rotor in a Heraeus Multifuge X1R (Thermo Fisher Scientific) centrifuge to obtain the supernatant, which was frozen until further use.

### Western blotting analyses

For Western blotting, cells were trypsinized for 2 min with TrypLE (Gibco) and collected by centrifugation at 2000×*g* for 10 min. The cell pellets were lysed using lysis buffer (150 mM NaCl, 50 mM Tris–HCl pH 7.6, 0.5% Sodium deoxycholate, 1% Triton X100, 2% Sodium dodecyl sulfate (SDS), and 10 µL/mL Halt Protease Inhibitor Cocktail (Termo Fisher Scientific)) and briefly sonicated. Samples were diluted 1:4 with 4X Laemmli buffer (250 mM Tris-HCl pH6.8, 40% glycerol, 8% SDS, and Bromophenol blue) and 1% 2.5 mM Dithiothreitol (DTT) was added. After boiling the samples at 95 °C for 5 min, they were equally loaded onto a 4–20% Novex™ Tris-Glycine Mini Protein gel (Invitrogen) run at 90 V. The gel was subsequently transferred onto an iBlot2 Transfer Stack (Invitrogen) nitrocellulose membrane, using the iBlot2 machine (Invitrogen), followed by 1 h blocking in 2% non-fat milk (Semper). The membranes were then incubated at 4 °C overnight with primary antibodies. The primary antibodies used included: anti-HRS (1:400, Santa Cruz Biotechnology), anti-Tsg101 (1:1000, Abcam), anti-Vps25 (1:400, Santa Cruz Biotechnology), anti-Vps25 (1:1000, Invitrogen), anti-Chmp2a (1:1000, Proteintech), anti-nSmase2 (1:200, Santa Cruz Biotechnology), anti-Rab31 (1:1000, Novus Biologicals), 4H11 (1:500, as previously described [32]), anti-CD81 (1:500, Santa Cruz Biotechnology), anti-Alix (1:500, Cell Signaling), anti-Calreticulin (1:1000, Novus Biologicals), and anti-Aβ 6E10 (1:1000, Biolegend). Membranes were cut in half and probed with one antibody with suitable molecular weight of target protein. After the membranes were washed 3 × 5 min with TBS-Tween (Medicago), the secondary antibodies, goat-anti-mouse and goat-anti-rabbit (1:2000, DAKO), were incubated for 1.5 h at room temperature. After several washes, the membranes were visualized using SuperSignal West Femto Maximum Sensitivity substrate (Thermo Scientific) with a Chemidoc XRS + (Bio-Rad). Western blot membranes were re-probed with anti-β-Actin (1:10 000, Sigma Aldrich) for 1 h, followed by the same washing and secondary antibody steps. Densitometric analyses were performed using ImageJ2 and obtained values were normalized to β-Actin. Molecular weight of protein bands was analyzed using Image Labs 6.1, in relation to a Precision Plus Protein™ Dual Xtra Standard (Bio-Rad).

### Dot blotting analyses

For dot blotting, 2 µL cell medium or EV sample was dotted onto a nitrocellulose membrane (Bio-Rad). The membrane was allowed to air-dry completely for 20 min at room temperature prior to blocking with 2% non-fat milk (Semper). The membranes were then incubated at 4 °C overnight with primary antibody anti-Aβ 6E10 (1:1000, Biolegend). After the membranes were washed 3 × 5 min with TBS-Tween (Medicago), the secondary antibodies, goat-anti-mouse and goat-anti-rabbit (1:2000, DAKO), were incubated for 1.5 h at room temperature. After several washes, the membranes were visualized using SuperSignal West Femto Maximum Sensitivity substrate (Thermo Scientific) with a Chemidoc XRS + (Bio-Rad). Densitometric analyses were performed using ImageJ2.

### Semi-quantitative real-time PCR

Total RNA was isolated using Purelink™ RNA Mini Kit (Invitrogen) and then converted to cDNA using the High-Capacity cDNA Reverse Transcription Kit (Applied Biosystems), following manufacturer’s instructions. The cDNA was diluted to 5 ng/µL before performing semiquantitative real-time PCR (RT-PCR) using Powertrack™ SYBR Green Master Mix (Applied Biosystems™), according to manufacturer’s instructions. All primers were designed using the primer-design tool primer-BLAST (https://www.ncbi.nlm.nih.gov/tools/primer-blast/) or sourced from previous literature (Supplementary Table S1). Reactions were analyzed on a CFX96 Touch RT-PCR Detection System (Bio-Rad) and quantified using the comparative Ct method. Samples were normalized to β-actin and presented as ΔΔCt.

### Extracellular vesicle isolation

To isolate EVs, cell medium was thawed and centrifuged at 10 000xg for 35 min using a Sigma Fixed Angle Rotor 12,156-H (Sigma) in a Sigma 3-30KS (Sigma) centrifuge. The supernatant was further centrifuged at 40 000 RPM (~ 118 000xg) for 120 min using a Ti70 rotor in an Optima L-80 XP (Beckman Coulter) centrifuge. After discarding the supernatant, the pellet was washed in 20 mL cold PBS and centrifuged again for 120 min. Following this, the pellet was collected in ~ 500 µL PBS and stored at -20 °C until further analysis.

### Extracellular vesicle characterization

EVs were characterized by the presence of the EV-related markers (Tsg101, Alix and CD81) and the absence of cellular marker Calreticulin using Western Blot analysis, morphology by Transmission Electron Microscopy (TEM), and size and concentration by nanoparticle tracking analysis. Prior to Western blot and TEM analyses, the samples were further concentrated using Amicon Ultra 3 K filters (Millipore). For Western blot, 2 µg total protein was loaded for the cell control, and approximately a third of total EVs, isolated from 25 mL medium, was loaded onto the gel. For TEM, carbon-coated copper grids were coated with 5 µL sample for 10 min at room temperature. The excess liquid was removed and grids were washed twice with deionized water prior to negative staining with 2% uranyl acetate for 30 s. Samples were imaged with a JEM-1230-EX electron microscope (Jeol). Size and concentration measurements of EVs were obtained using a Nanosight ns300 (Malvern Panalytical) with a camera setting of 16 and detection threshold of 5. The results were normalized to account for variations in corresponding cell numbers.

### Human Aβ42 enzyme-linked immunosorbent assay (ELISA)

The Aβ content of the EVs was quantified using the Human βAmyloid (1–42) ELISA Kit *Wako*, High sensitivity (Fujifilm). Following Amicon Ultra 3 K filtration (Millipore), EV samples were diluted 19:1 with lysis buffer, followed by a 1:4 dilution with sample diluent from the kit. To lyse the EVs further, the sample was sonicated in a water bath for 2 min, followed by 2-min incubation on ice. Subsequently, 100 µL sample was loaded into each well, and the remaining protocol was performed per manufacturer’s instructions. The absorbance was read using a Spark 10 M (Tecan) microplate reader at 450 nm. The fM concentration of Aβ in the sample was normalized to EV amount.

### Statistical analyses

All data were analyzed using suitable statistical comparisons in GraphPad Prism 9. Normal distribution of the data was assessed by evaluation of a normal probability plot and Shapiro–Wilk test, and depending on the number of groups, either a Student’s t-test (for two group comparisons) or a one-way ANOVA followed by Tukey’s post-hoc test (for comparisons involving three or more groups) was conducted. A two-way ANOVA followed by a Fisher’s LSD test was performed when comparing the effects of two variables (cell type and treatment). Prior to statistical analysis, outliers in the dataset were evaluated using the GraphPad Prism ROUT method and those verified as valid data points were retained. Data are presented as mean ± SD. Specific details regarding statistical analysis for each experiment are provided in respective figure legends. Inkscape was used for compilation of figures.

## Results

### Stable overexpression of PrP^C^ affects Chmp2a, Vps25, and Rab31 protein levels in cells

**Fig. 1:**
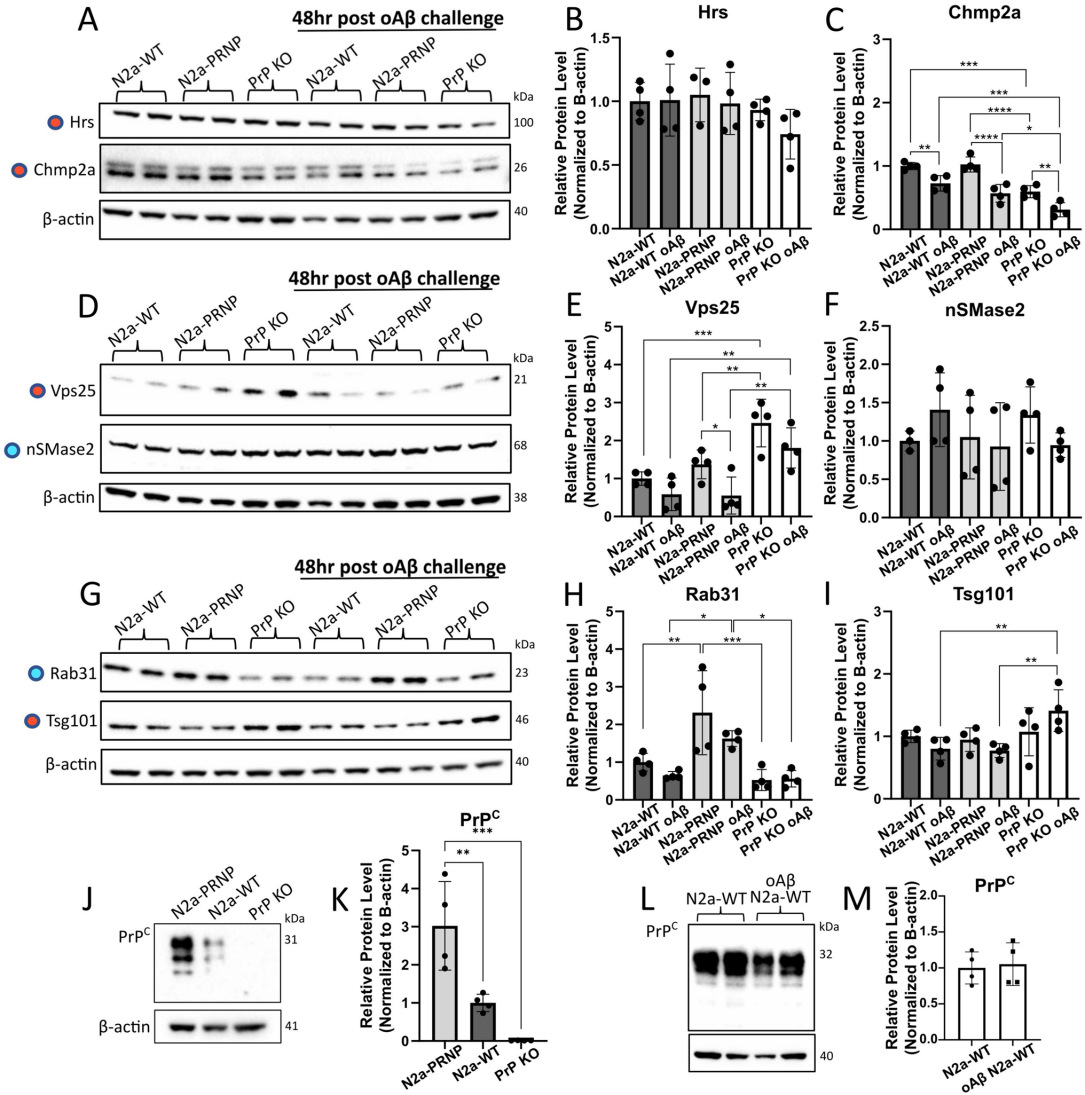
oAβ treatment affects exosome biogenesis-related proteins. N2a-WT, N2a-PRNP, and PrP KO cells were treated with 2.5 µM oAβ for 6 h, followed by 48-h incubation in fresh medium without oAβ. This led to **A**–**I** changes in several exosome biogenesis-related protein levels, demonstrated by Western blot quantification. ESCRT-dependent proteins are marked with a red dot and ESCRT-independent proteins are marked with a blue dot. **J** and **K** N2a-PRNP showed an overexpression of PrP^C^ compared to N2a-WT and PrP KO. **L** and **M** oAβ challenge did not affect PrP^C^ levels in N2a-WT cells. *p < 0.05, **p < 0.01, ***p < 0.001, ****p < 0.0001, two-way ANOVA with Fisher’s LSD test. **p < 0.01, ***p < 0.001, one-way ANOVA with Tukey post-hoc test. *n* = 4 independent biological replicates. Data presented as mean ± SD

Since different exosome biogenesis-related proteins have previously shown to be involved in various outcomes of exosome output, we assessed the effect of PrP^C^ overexpression and deletion on different proteins involved in each ESCRT subcomplex in cells (hepatocyte growth factor-regulated tyrosine kinase substrate (Hrs), tumor susceptibility gene 101 (Tsg101), vacuolar protein sorting 25 (Vps25), charged multivesicular body protein 2A (Chmp2a)), and two proteins from the ESCRT-independent pathway (neutral sphingomyelinase 2 (nSMase2) and Rab31). We compared these exosome biogenesis-related protein levels in the stable PrP^C^-overexpressing N2a-PRNP cells, and transiently PrP^C^-transfected PrP KO cells (PrP KO^OE^), to N2a-WT and PrP KO cells using Western blot analysis. Our results revealed similar protein levels for Hrs, nSMase2, and Tsg101 between all cell types (Fig. 1A, B, D, F, G, I; Supplementary Fig. 1B, C, E, G, H, J). Conversely, Chmp2a was significantly decreased in PrP KO cells compared to both N2a-WT and N2a-PRNP cells (Fig. 1A, C), but this effect was not rescued by transient transfection in the PrP KO^OE^ cells (Supplementary Fig. 1B, D). Again, a significant increase of Vps25 was identified in PrP KO cells compared to N2a-WT, N2a-PRNP, and PrP KO^OE^ cells (Fig. 1D, E; Supplementary Fig. 1E, F). As opposed to transient transfection, the N2a-PRNP cells demonstrated a significantly increased Rab31 protein level compared to both N2a-WT and PrP KO cells (Fig. 1G, H). Thus, stable overexpression of PrP^C^ confirmed differences seen with transient PrP^C^ overexpression (Vps25), but also uncovered new effects (Chmp2a, Rab31). These differences might be caused by differences in PrP^C^ expression levels (Fig. 1JK; Supplementary Fig. 1A), time of expression, or other factors between transient transfection and stable transfection. Further mRNA analysis demonstrated minimal biological differences for the analyzed proteins, but with no corresponding change at protein level (Supplementary Fig. 2). Thus, further investigations were done at protein level only.

### oAβ impacts exosome biogenesis-related proteins in cells

To investigate the additive effect of oAβ on exosome biogenesis-related proteins, we challenged the N2a-WT, N2a-PRNP, and PrP KO cells with 2.5 µM oAβ for 6 h, followed by a media change and continued incubation without oAβ for 48 h. To assess the early effect of oAβ, we first conducted Western blot analyses directly after a 6 h oAβ challenge. At this timepoint, we observed no significant additional effect of oAβ for Hrs, Chmp2a, Vps25, or nSMase2 (Supplementary Fig. 3A–F). However, Rab31 protein levels were significantly increased in N2a-PRNP cells after 6 h oAβ challenge compared to treated N2a-WT and PrP KO cells (Supplementary Fig. 3G, H). Nonetheless, this effect could be explained by a general increase of Rab31 levels in N2a-PRNP cells. In addition, Tsg101 protein levels were significantly increased in untreated PrP KO cells, while oAβ challenge caused a significant decrease of these levels (Supplementary Fig. 3G, I). After media change and continued incubation without oAβ for 48 h, Western blot analysis showed no effect in Hrs cellular protein levels (Fig. 1A, B). However, we observed significantly decreased Chmp2a levels in all cell types upon oAβ treatment compared to the respective untreated controls (Fig. 1A, C). At 48 h, the effect of oAβ challenge was more apparent, showing decreased Vps25 levels in all cell types, with a significant decrease in N2a-PRNP cells and a strong trend in PrP KO cells (*p* = 0.058) (Fig. 1D, E). nSMase2 and Rab31 showed similar results at both 6 h and 48 h (Fig. 1D, F–H). Interestingly, oAβ challenge caused significant increased Tsg101 levels in PrP KO cells compared to both N2a-WT and N2a-PRNP cells (Fig. 1G, I). The effects of oAβ were not caused through changes to PrP^C^ levels as these were unchanged in N2a-WT cells at both 6 and 48 h (Supplementary Fig. 3 J, K; Fig. 1L, M). Thus, we conclude that oAβ challenge resulted in significantly decreased levels of ESCRT-dependent proteins Chmp2a and Vps25, potentially leading to a decreased ESCRT-dependent exosome biogenesis. Additionally, the increased level of ESCRT-dependent protein Tsg101, in only PrP KO cells, further emphasizes the impact of PrP^C^ on exosome biogenesis. Taken together, these data show that oAβ changes the exosome biogenesis protein levels in a PrP^C^-dependent manner.

### Serum-free media significantly change exosome biogenesis-related proteins, cell proliferation, and EV abundance

Regular fetal bovine serum (FBS) medium contains high amount of EVs and cannot be used for EV research, while serum withdrawal promotes differentiation of N2a cells through pathways potentially also activating the ESCRT-dependent pathway [33–35]. Thus, we compared the effect of 48 h serum withdrawal on cellular levels of selected exosome biogenesis-related proteins compared to media supplemented with regular FBS or a commercial EV-depleted FBS (Gibco). Several changes were detected between serum-free medium and the EV-depleted FBS (Supplementary Fig. 4), but only nSMase2 protein levels in PrP KO cells were significantly increased in serum-free medium compared to regular FBS (Supplementary Fig. 4E, H). As EV output largely depends on number of cells, we determined the media effect on cell count. Cell concentration in serum-free medium and EV-depleted FBS was about half of that in regular FBS after 48 h, with no significant difference in cell viability detected using Trypan blue stain (Supplementary Fig. 4 M, N). However, culturing cells in serum-free medium resulted in significantly higher numbers of EVs compared to EV-depleted FBS (Supplementary Fig. 4O, P). This is in line with previous reports on increased EV release from N2a cells upon serum withdrawal [36]. Hence, all further experiments were performed using EV-depleted FBS.

### PrP^C^ deletion results in intracellular Aβ accumulation and increased EV abundance

**Fig. 2:**
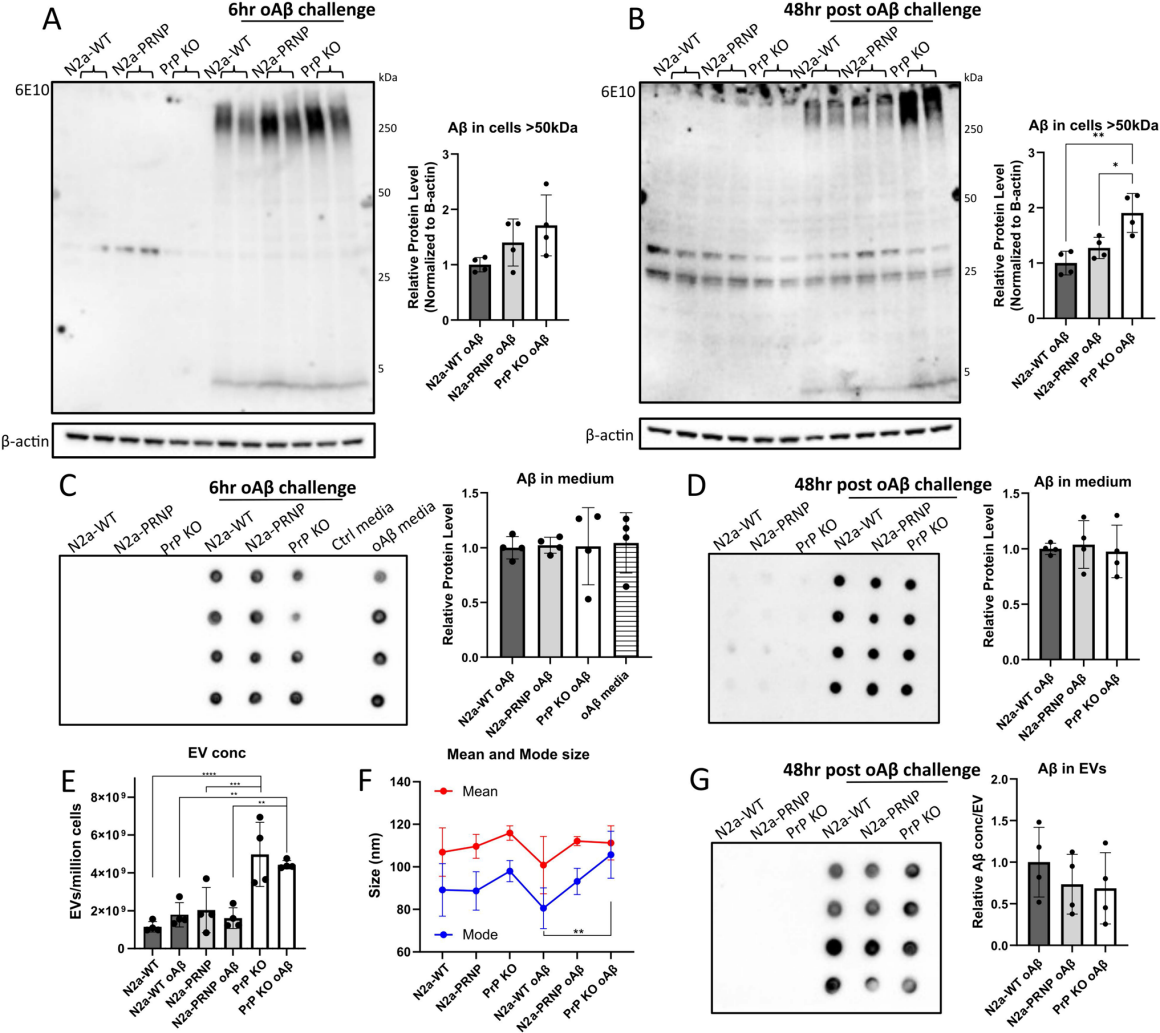
Lack of PrP^C^ causes accumulation of Amyloid β. Cells were treated for 6 h with 2.5 µM oAβ and fresh medium without oAβ was added for 48 h. **A** Western blot quantification showed a trend toward increased intracellular accumulation in PrP KO cells at 6 h and **B** a significant increased level at 48 h. **C** and **D** Dot blot quantification showed no change in Aβ level in the medium at either 6 or 48 h. **E** EVs were isolated from 48 h cell medium and quantified using NTA analysis. PrP KO EVs showed a higher abundance of particles compared to both N2a-WT and N2a-PRNP EVs. **F** Mean size of EVs did not differ, while mode size was significantly different between oAβ-treated PrP KO cells and oAβ-treated N2a-WT cells. **G** Dot blot quantification showed no significant difference in Aβ content of the EVs. *p < 0.05, **p < 0.01, one-way ANOVA with Tukey post-hoc test. **p < 0.01, ***p < 0.001, ****p < 0.0001, two-way ANOVA with Fisher’s LSD test. *n* = 4 independent biological replicates. Data presented as mean ± SD

Having seen an impact on exosome biogenesis-related proteins in cells, upon both PrP^C^ overexpression and oAβ challenge, we next investigated whether PrP^C^ is involved in the release of oAβ via exosomes. There are several practical limitations to using transient transfections to study EVs including loss of plasmid and reduced expression over time. Therefore, we continued to utilize the stable overexpressing N2a-PRNP cell line. Thus, we challenged N2a-WT, N2a-PRNP, and PrP KO cells with 2.5 µM oAβ for 6 h. Comparative analysis of Aβ accumulation at 6 h showed a tendency of increased cellular Aβ levels in N2a-PRNP and PrP KO cells, relative to N2a-WT, as measured by Western blot analysis (Fig. 2A). After media change and continued incubation without oAβ for 48 h, PrP KO cells showed significantly higher Aβ levels (Fig. 2B). However, no differences were observed in Aβ levels in the media across these cell types and timepoints, as determined using dot blot analysis (Fig. 2C, D). Hence, the intracellular Aβ accumulation was likely not due to a change in cellular uptake of oAβ or general release, but rather to a slower removal or processing of oAβ, explaining the lack of differences in Aβ present in total media.

To further investigate whether Aβ is released in EVs differently between N2a-WT, N2a-PRNP, and PrP KO cells, we then isolated EVs from the 48 h cell medium, and characterized them according to the 2018 MISEV guidelines [37]; detecting relevant protein markers by Western blot analysis (positive for Tsg101, Alix, and CD81; negative for Calreticulin), size determination by Nanoparticle tracking analysis (NTA), and morphology by Transmission electron microscopy (TEM) (Supplementary Fig. 5). NTA showed significantly higher EV abundance released from PrP KO cells (Fig. 2E; Supplementary Fig. 5B). Though oAβ challenge did not affect the amount of EVs released from any of the cell types analyzed (Fig. 2E), it did result in a significant difference in EV mode size between N2a-WT and PrP KO cells (Fig. 2F). This could indicate a difference in EV structure or amount of cargo, although dot blot analysis did not detect differences in EV Aβ levels (Fig. 2G).

### Inhibition of nSMase results in intracellular Aβ accumulation and abolished Aβ EV content

**Fig. 3:**
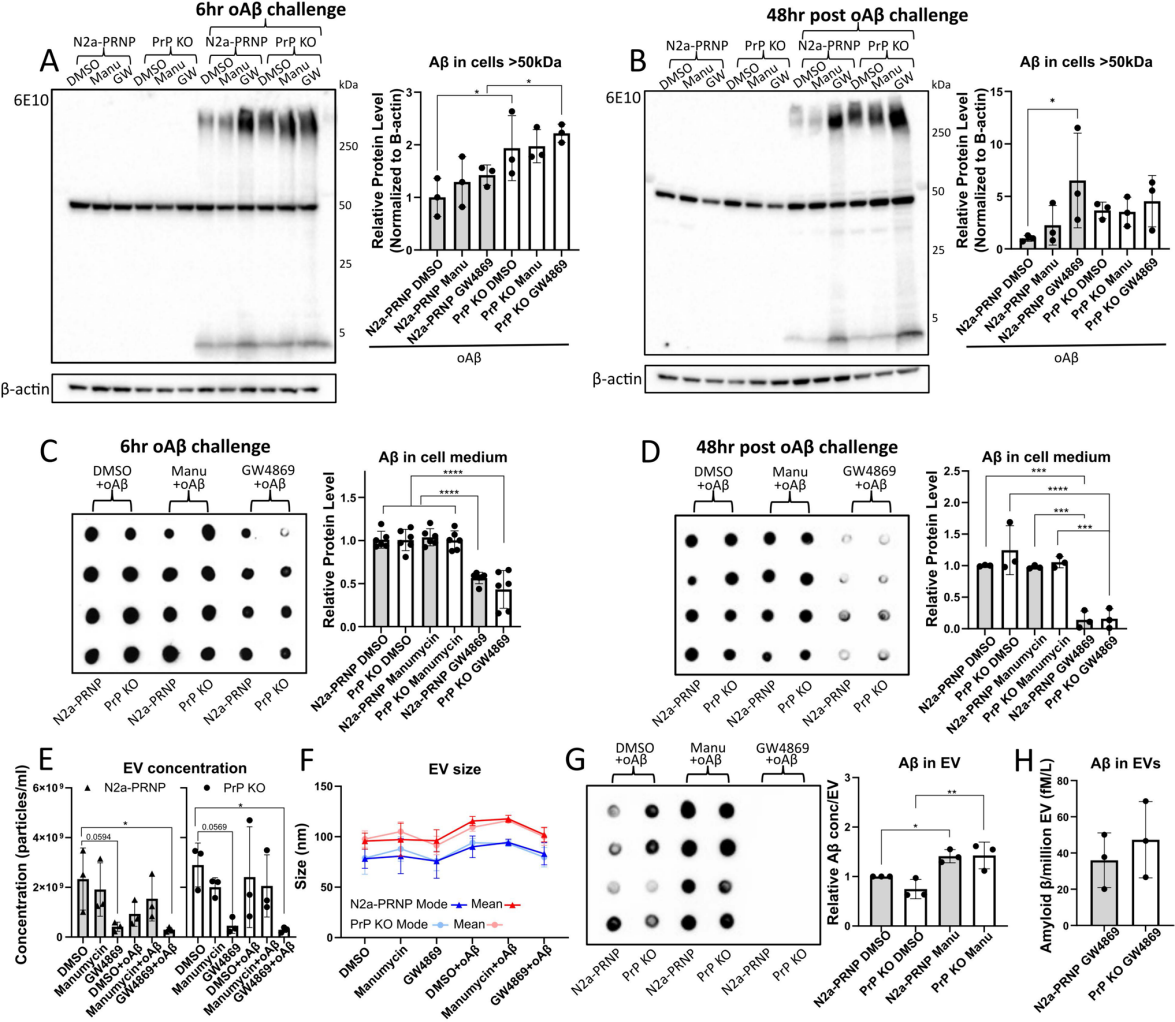
Amyloid β content in cells and EVs are affected by exosome inhibitors. Cells were treated for 6 h with 2.5 µM oAβ and exosome inhibitors Manumycin A (Manu) and GW4869 (GW) or DMSO control and fresh medium with drugs but without oAβ was added for another 48 h. **A** Western blot quantification showed a significant increased intracellular Aβ accumulation in PrP KO cells at 6 h. **B** At 48 h, Western blot quantification showed only a significant higher intracellular Aβ accumulation in N2a-PRNP cells followed by GW4869 treatment. **C** and **D** Dot blot quantification showed a significant decrease of Aβ in GW4869 cell medium both at 6 and 48 h. **E** EVs were isolated from 48 h cell medium and quantified using NTA analysis. Both inhibitors demonstrated trends of decreasing EV abundance, with a significant decrease upon oAβ together with GW4869 treatment, for both cells. **F** Neither mean size nor mode size of EVs was significantly different upon oAβ and inhibitor treatment. **G** Dot blot quantification showed significant difference in Aβ content of the EVs with an increased concentration upon Manumycin A treatment and a non-detectable level upon GW4869 treatment. **H** When employing a high-sensitivity ELISA, no significant difference in Aβ level could be detected between N2a-PRNP and PrP KO EVs upon GW4869 treatment. *p < 0.05, **p < 0.01, ***p < 0.001, ****p < 0.0001, two-way ANOVA with Fisher’s LSD test, *n* = 3 independent biological replicates. Data presented as mean ± SD

To assess whether oAβ secretion utilizes a specific exosome pathway and if this is dependent on PrP^C^, we inhibited the exosome biogenesis pathways in both N2a-PRNP and PrP KO cells using either the ESCRT-inhibitor Manumycin A or the ESCRT-independent (nSMase) inhibitor GW4869, in concentrations previously reported to decrease EV release (for example in [38, 39]) and confirmed not to affect cell viability in the current experimental settings (data not shown). PrP KO cells showed increased cellular Aβ accumulation at 6 h (Fig. 3A), without any additional effect upon exosome inhibition. However, 48-h nSMase inhibition led to significant Aβ accumulation in N2a-PRNP cells using Western blot analysis (Fig. 3B). Importantly, decreased levels of Aβ in cell medium were observed in both cell types at 6 h and 48 h as analyzed by dot blot (Fig. 3C, D). NTA analysis showed a strong trend for decreased EV abundance in conditioned medium after nSMase inhibition in both N2a-PRNP (*p* = 0.0594) and PrP KO cells (*p* = 0.0569), and combined with oAβ challenge resulted in a significant decrease in both cell types (Fig. 3E). However, no significant difference was observed for Manumycin A treatment, which could potentially be due to a compensatory mechanism of EV release via the opposite pathway to the one being inhibited, as previously suggested for tetraspanins [40]. To confirm that Manumycin A still inhibited the ESCRT-derived exosomes, we demonstrated a decreased level of Hrs, Tsg101, and Alix in the EVs by dot blot (Supplementary Fig. 6). No changes in EV size were detected between treatments (Fig. 3F). To establish the Aβ content of EVs, isolated vesicles were analyzed using dot blot showing significantly increased levels of Aβ in EVs isolated from Manumycin A-treated cells (Fig. 3G). On the other hand, EVs isolated from GW4869-treated cells showed undetectable levels of Aβ. A high-sensitivity ELISA could detect low levels of Aβ in these EVs from GW4869-treated cells, from both cell types but with no significant difference (Fig. 3H). These results demonstrate that Aβ is mainly released via an ESCRT-independent pathway, and a potential compensatory effect results in increased Aβ release when inhibiting the ESCRT-dependent pathway.

### Exosome inhibitors affect cellular levels of PrP^C^ and exosome biogenesis-related proteins

**Fig. 4:**
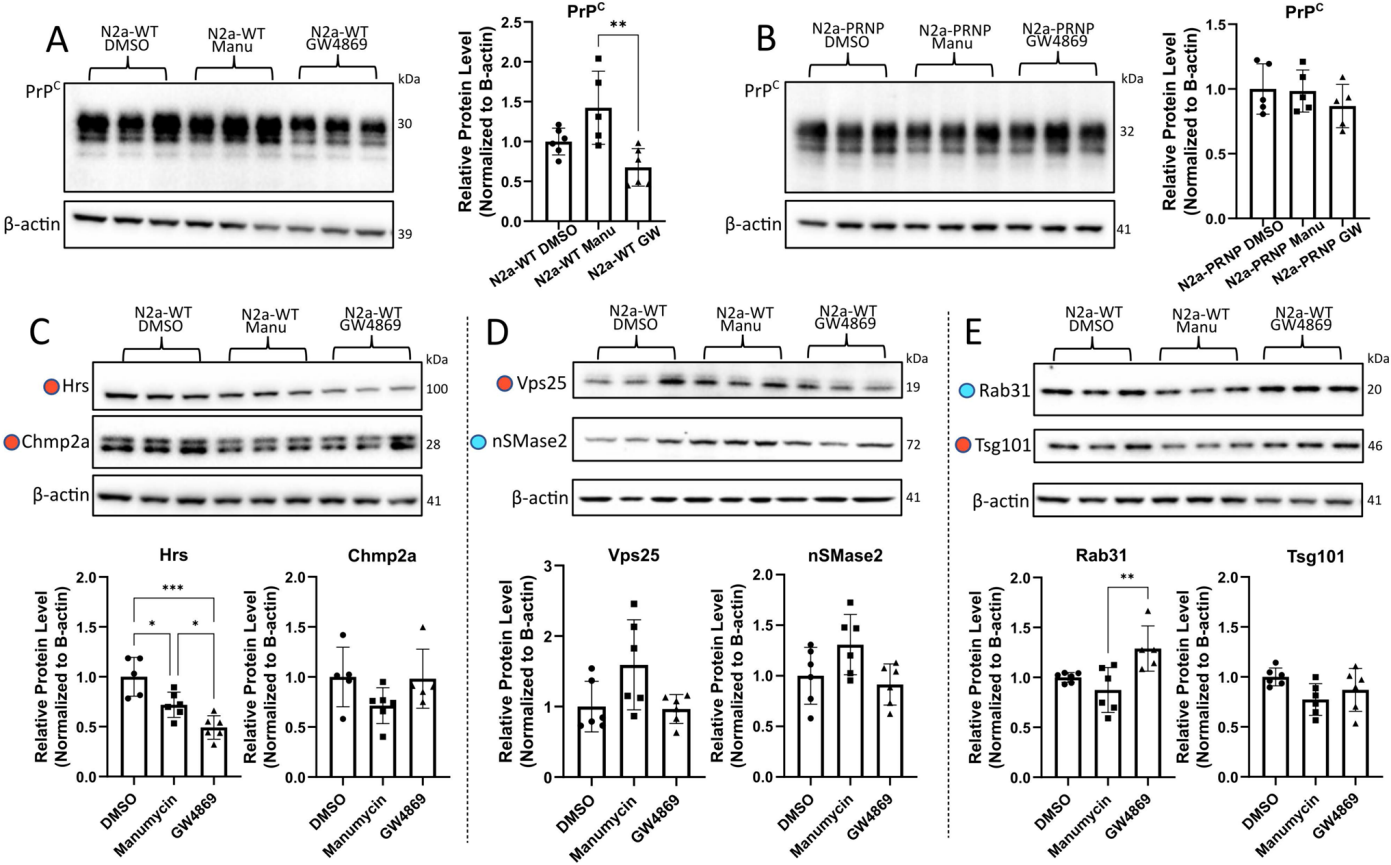
Exosome inhibitors affect level of exosome biogenesis-related proteins and PrP^C^. 48-h treatment with exosome inhibitors Manumycin A (Manu) and GW4869, or DMSO control, resulted in **A** a significant difference in PrP^C^ level between Manumycin A- and GW4869-treated N2a-WT cells, demonstrated by Western blot quantification. **B** This difference was abolished in the N2a-PRNP cells. **C**–**E** Several exosome biogenesis-related proteins were also affected differently by the two exosome-inhibitor treatments. ESCRT-dependent proteins are marked with a red dot and ESCRT-independent proteins are marked with a blue dot. *p < 0.05, **p < 0.01, one-way ANOVA with Tukey post-hoc test, *n* = 5–6 independent biological replicates. Outliers and misshapen Western blot bands were excluded from the analysis. Data presented as mean ± SD

Since N2a-PRNP cells both showed an increased Aβ accumulation upon GW4869 treatment (Fig. 3B) and released a significantly lower amount of EVs compared to PrP KO cells (Fig. 2E), we investigated if the inhibitors had a direct effect on PrP^C^ in cells. Western blot analysis showed that treatment with Manumycin A induced significantly higher intracellular PrP^C^ levels compared to GW4869 treatment in N2a-WT cells (Fig. 4A). As expected, no difference could be detected in N2a-PRNP cells upon drug treatment (Fig. 4B), probably due to the overexpression of PrP^C^ in these cells. Surprisingly, GW4869 treatment significantly reduced the cellular levels of the ESCRT-dependent protein Hrs compared to DMSO control, with a lesser decrease after Manumycin A treatment (Fig. 4C). No significant changes were noted for Chmp2a, Vps25, Tsg101, and nSMase2 upon treatment with either of the exosome inhibitors (Fig. 4C–E). For the ESCRT-independent protein Rab31, GW4869 treatment resulted in significantly higher levels compared to Manumycin A treatment (Fig. 4E). Thus, the exosome inhibitors only influence the protein level of some of the investigated exosome biogenesis-related proteins, Hrs and Rab31. Importantly, we demonstrated that GW4869 influences the protein levels of the ESCRT-dependent pathway to some degree, a result not previously reported with tremendous implications for EV research.

We then conducted similar analyses in N2a-PRNP and PrP KO cells to investigate whether the changes in exosome biogenesis-related proteins upon exosome inhibition were dependent on PrP^C^. Similar to N2a-WT cells, GW4869 treatment resulted in significant decreased levels of Hrs and significantly increased levels of Rab31 compared both to DMSO controls and Manumycin treatment in N2a-PRNP cells (Supplemental Fig. 7A, B, G, H). Importantly, this effect was not demonstrated in PrP KO cells. Instead, Manumycin A treatment caused a significant increase in nSMase2 level in PrP KO cells compared to DMSO control and GW4869 treatment (Supplemental Fig. 7D, F). Thus, the exosome inhibitors only influence the protein level of some of the investigated exosome biogenesis-related proteins. Previously not described, however, is that Manumycin A and GW4869 have an influence on the protein levels of the opposite exosome biogenesis pathway, a mechanism dependent on the presence of PrP^C^, further supporting the influence of PrP^C^ on exosome biogenesis.

## Discussion

In this study, we explored the role of the PrP^C^ protein on the release of oAβ through exosomes, revealing several novel insights into the molecular mechanisms underpinning AD pathogenesis. Firstly, we demonstrate that lack of PrP^C^ result in an increase of the exosome biogenesis-related protein Vps25 and an increased EV abundance. These results are critical in understanding the cellular response and spreading of oAβ in AD, particularly noting the increase in Aβ accumulation in cells lacking PrP^C^. Still, PrP^C^ did not affect Aβ levels in EVs, nor did it affect exosome biogenesis pathway through which the Aβ was released. Secondly, oAβ challenge significantly reduced Chmp2a and Vps25 levels, independent of PrP^C^. However, this did not translate into changes in EV abundance. Lastly, the inhibition of exosome pathways revealed a preferential release of Aβ via a proposed ESCRT-independent pathway, a process seemingly unaffected by PrP^C^. The application of exosome inhibitors further highlighted a significant decrease in Hrs for both Manumycin A and GW4869 and opposite effects for Rab31, dependent on the presence of PrP^C^. Furthermore, the levels of PrP^C^ were also significantly higher after Manumycin A treatment compared to GW4869 treatment. These results contribute to the hypothesis that PrP^C^ modulates exosome biogenesis and highlights the importance of the preferential incorporation of Aβ into GW4869-specific exosomes.

Previous studies have shown that PrP^C^ deficiency causes a proteome shift in various cell models, including N2a [28], and PrP^C^ expression has been correlated with EV release in astrocytes and fibroblasts [22]. Aligning with these studies, our results demonstrate an increase in Vps25 upon PrP^C^ deletion. However, our observed increase in EV abundance in PrP^C^-lacking cells contrasts with these previous findings [22], suggesting either potential cell type-specific differences or more likely depends on variances arising from methodological approaches in quantifying EVs. This emphasizes the importance of relating EV abundance to the cell type and cell density at harvest, as recommended by the 2018 MISEV guidelines [37].

Challenging PrP^C^-deficient cells with oAβ caused intracellular accumulation of Aβ but with no change in media or EV levels of Aβ, indicating that cells containing PrP^C^ degraded the oAβ more efficiently. This pattern aligns with previous research indicating PrP^C^’s role in autophagy activation in general [41] and in response to Aβ [42]. An impaired autophagy has also shown to cause intracellular Aβ accumulation specifically in the Golgi and a subsequent decrease in MVBs [43], suggesting the importance of further studies focusing on differences in autophagy function in relation to Aβ accumulation, PrP^C^ expression, and EVs. Furthermore, the unchanged Aβ levels in EVs across cell models, despite varying cellular concentrations, confirm that the EV cargo does not solely mirror cellular levels. Although oAβ had no direct impact on EV abundance, we show for the first time that oAβ significantly influenced several exosome biogenesis-related proteins including the decreased levels of Vps25 and Chmp2a. Notably, previous research has shown *CHMP2A* to be increased by age in healthy brains while reduced in AD brains [15] and lack of *CHMP2A* promotes tau aggregation and endolysosomal leakiness [44]. Our results also demonstrated a PrP^C^-dependent decreased level of Tsg101 upon oAβ challenge. As Tsg101, Vps25, and Chmp2a have been described to have additional cellular functions, including autophagy functions [45–48], it suggests that oAβ treatment and PrP^C^ levels may influence not just exosome biogenesis but also other critical cellular pathways implicated in AD.

Despite GW4869 and Manumycin A being widely used drugs in exosome research, no previous study has, to our knowledge, investigated the cross-over effect of these drugs on exosome biogenesis-related proteins involved in the opposite exosome pathways. Our investigation revealed a significant decrease in Hrs for both inhibitors, a previous unknown effect of GW4869 treatment but a suggested effect of Manumycin A treatment [38]. Hrs silencing has previously shown to target sphingomyelin to lysosomes [49] which open up a new connection between the two exosome biogenesis pathways. Whether this decrease in Hrs is an indirect effect of an increased level of sphingomyelin, as a result of the GW4869 treatment, or whether GW4869 acts directly on Hrs remains to be established. Moreover, while the GW4869 drug produced a similar effect independent on PrP^C^ expression, Manumycin A treatment did not result in a decrease of Hrs in either the PrP^C^-overexpressing cell or the knockout cell. Similar to previous studies [38], we confirmed that the Manumycin A treatment has an inhibitory function on ESCRT-dependent exosome biogenesis by a decrease in ESCRT-dependent protein levels in Manumycin A-treated EVs, from both PrP^C^-overexpressing cells and knockout cells. Thus, PrP^C^ appears to be important for intracellular Hrs levels. The PrP^C^ expression was also important for Rab31 level and nSMase2 level, as both were significantly increased in opposite cells upon either GW4869 or Manumycin A treatment, respectively. Furthermore, our results show that exosome inhibitors do not always reduce EV abundance in conditioned medium, only GW4869 treatment caused a strong trend of lowering EVs in PrP^C^-overexpressing cells and KO cells. This might be due to compensatory mechanisms in the opposite exosome pathway or PrP^C^-dependent mechanisms. Hence, further research investigating the interaction between the different exosome pathways is needed.

Importantly, GW4869 treatment in PrP^C^-overexpressing cells caused a significant increase in accumulation of cellular Aβ, potentially due to the significant decrease in Aβ released in EVs or the decrease in medium Aβ. Surprisingly, cells lacking PrP^C^ also displayed significantly reduced Aβ levels in EVs and medium, but not increased intracellular Aβ, upon GW4869 treatment. This could be explained by reduced degradation of oAβ in PrP^C^-overexpressing cells, due to suppressed autophagy, potentially caused by the combined effect of lower levels of Vps25 and Tsg101 [46, 48], together with a suppressed autophagy by GW4869 treatment [50]. The increased level of Aβ content in EVs after Manumycin A treatment is likely a result of a compensatory mechanism from the ESCRT-independent pathway, which further emphasizes the importance of this pathway in aggregated Aβ release as previously suggested [16]. Interestingly, inhibition with Manumycin A and GW4869 also resulted in opposite effects on PrP^C^ level in cells, with significant decreased PrP^C^ level upon GW4869 treatment compared to Manumycin A treatment, strengthening previously reported trends [20]. This could potentially be due to a decreased degradation of PrP^C^, an inhibited release of PrP^C^ via the ESCRT-dependent pathway, or an upregulated PrP^C^ production. Taken together, this implies a complex interplay between the different exosome biogenesis pathways and emphasizes the potential of PrP^C^ in these mechanisms.

In conclusion, our study provides compelling evidence for the integral role of PrP^C^ in exosome biogenesis and oAβ release, highlighting its significance in AD progression. Notably, lack of PrP^C^ resulted in Aβ accumulation, increased EV abundance, and changes in exosome biogenesis-related proteins Vps25, Chmp2a, and Rab31, accentuating its important role in AD pathogenesis and basal exosome biogenesis. The interplay between PrP^C^ and exosome biogenesis is also highlighted by the inverse regulation of PrP^C^ levels upon inhibition of the opposite exosome biogenesis pathways. Furthermore, the importance of exosomes in the spread of toxic Aβ is emphasized by the clear reduction in Aβ release upon GW4869 treatment, proposedly by inhibiting the ESCRT-independent pathway. Despite the attractiveness of inhibiting this pathway to slow down the propagation of AD, our results emphasize the importance in first understanding the basic functions of these multifaceted cellular mechanisms due to potential risk of cross-over effects. Overall, these insights underline the importance of PrP^C^ and exosome biogenesis-related proteins in AD progression and offers valuable perspectives for future therapeutic interventions targeting exosome-mediated pathways in AD.

## Supplementary information

This article contains supplementary information with supplementary figures and details of primer sequences designed using Primer-BLAST (https://www.ncbi.nlm.nih.gov/tools/primer-blast/) or sourced from previous literature [51–54].

## Supplementary Information

Below is the link to the electronic supplementary material.

Supplementary file1 (DOCX 2207 kb)

Supplementary file2 (PDF 4486 kb)

## Data Availability

All relevant data have been presented in this manuscript or supplementary information in ‘Supporting information 1,’ for supplementary figures, and ‘Supporting information 2,’ for original Western blots. Further clarification of results will be provided upon request from corresponding author.
