# Supplementary material for: Lack of cellular prion protein causes Amyloid β accumulation, increased extracellular vesicle abundance, and changes to exosome biogenesis proteins
Source: Mol Cell Biochem. 2024 Jul 6;480(3):1569–82. doi: 10.1007/s11010-024-05059-0 (PMC11842432; doi:10.1007/s11010-024-05059-0)
Supplement: Supplementary file 1 — Supplementary file1 (DOCX 2207 kb) [file 11010_2024_5059_MOESM1_ESM.docx]

**Lack of cellular prion protein causes Amyloid β accumulation, increased extracellular vesicle abundance, and variations in exosome biogenesis proteins**

Lovisa Johansson^1*^, Juan F. Reyes^1^, Tahir Ali^2,3^, Hermann Schätzl^2,3^, Sabine Gilch^2,3^, Martin Hallbeck^1*^

^1^Department of Biomedical and Clinical Sciences and Department of Clinical Pathology, Linköping University, Linköping, Sweden.

^2^ Calgary Prion Research Unit, Faculty of Veterinary Medicine, University of Calgary, Calgary, Alberta, Canada.

^3^ Hotchkiss Brain Institute, University of Calgary, Calgary, Alberta, Canada.

*Corresponding authors

Corresponding authors’ emails: [Lovisa.Johansson@liu.se](mailto:Lovisa.Johansson@liu.se), [Martin.Hallbeck@liu.se](mailto:Martin.Hallbeck@liu.se)

**Supplementary Figures and Tables**

**Table S1. Primers used for semiquantitative qPCR.** Designed using Primer-BLAST (<https://www.ncbi.nlm.nih.gov/tools/primer-blast/>) or sourced from previous literature.

| Primer | Sequence | Source |
| --- | --- | --- |
| HRS | F: GAATGCCGTGAGCACTTTTGT  R: CCTCGTAGTACAGCCTGCG | Primer-BLAST |
| TSG101 | F: TGGCTGCTGGACACATACCC  R: GCAACTCTGACCGTGGATGTT | Primer-BLAST |
| VPS25 | F: GTTCCGGGTTTTCTGGGCTA  R: GTTAAGGGAATGGCGCTGTAG | Primer-BLAST |
| CHMP2a | F: GAAGCGGAAACCCATCGTTC  R: AGACCTCGATGTGGGTAGGG | Primer-BLAST |
| SMPD3 | F: GGGGTCTACGGTTGTCATGG  R: CTTCCCACCTGCACCTTGAG | Akeus et al. (2021) [1] |
| RAB31 | F: CGGGAGCTCAAAGTGTGTCT  R: CTGCAGCAGATCCTCGGTAG | Liang et al. (2019) [2] |
| PRNP | F: GTCGCATCGGTGGCAGGACT  R: CAGCCAGTAGCCAAGGTTCGCC | Kaczmarczyk et al. (2016) [3] |
| B-ACTIN | F: CTCAGGAGGAGCAATGATCTTGAT  R: TACCACCATGTACCCAGGCA | Torres et al. (2015) [4] |


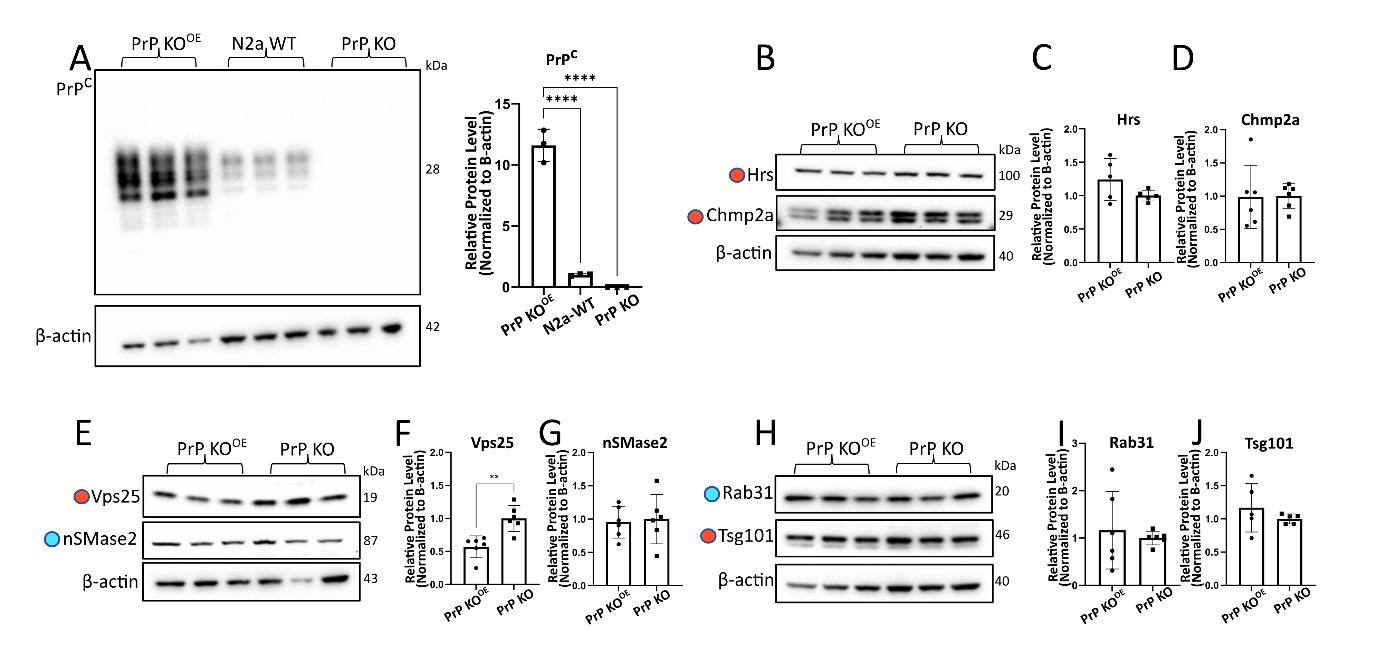


**Supplementary Figure 1. Transient PRNP transfection results in lower Vps25 protein levels.** A) PrP KO cells were transfected with plasmid vector (pcDNA3.1) expressing mouse 3F4-PrP (PrP KO^+OE^ cells) and showed high PrP^C^ level compared to N2a-WT and PrP KO cells, shown by Western blot quantification. For imaging purposes of PrP^C^, half the total protein concentration was loaded onto the gel for PrP KO^+OE^ cells in this blot. B-J) Western blot analyses for each protein of interest revealed only the ESCRT-dependent protein Vps25 to be significantly affected by PRNP transfection. ****p<0.0001, one-way ANOVA with Tukey post-hoc test. **p<0.01, Student’s T-test. N2a-WT n=3; PrP KO^OE^ and PrP KO n=5-6 independent biological replicates. Outliers and misshapen western blot bands were excluded from the analysis, including lane 5 of figure F. Data presented as mean±SD.


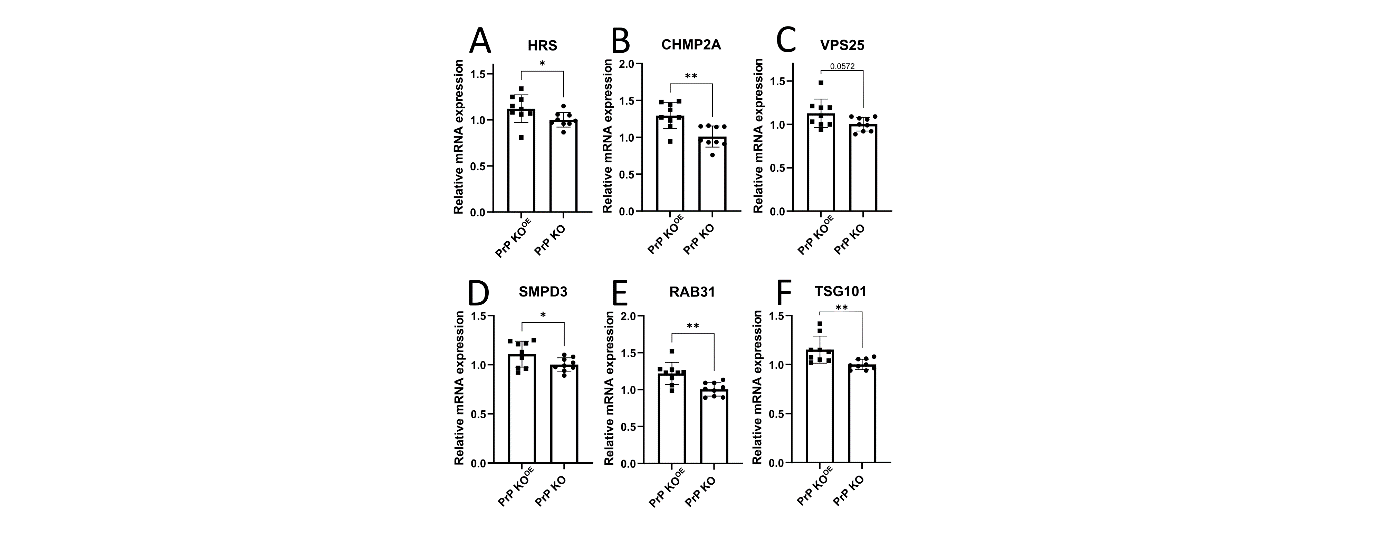


**Supplementary Figure 2. PRNP transfection has minimal effect on exosome biogenesis-related mRNA levels.** A-F) Transfecting PrP KO cells with 2ng plasmid vector (pcDNA3.1) expressing mouse 3F4-PrP resulted in small increases in mRNA level of most exosome-biogenesis related proteins, except for VPS25. *p<0.05, **p<0.01, Student’s t-test, n=9 independent biological replicates. Data presented as ΔΔCt values, mean±SD.


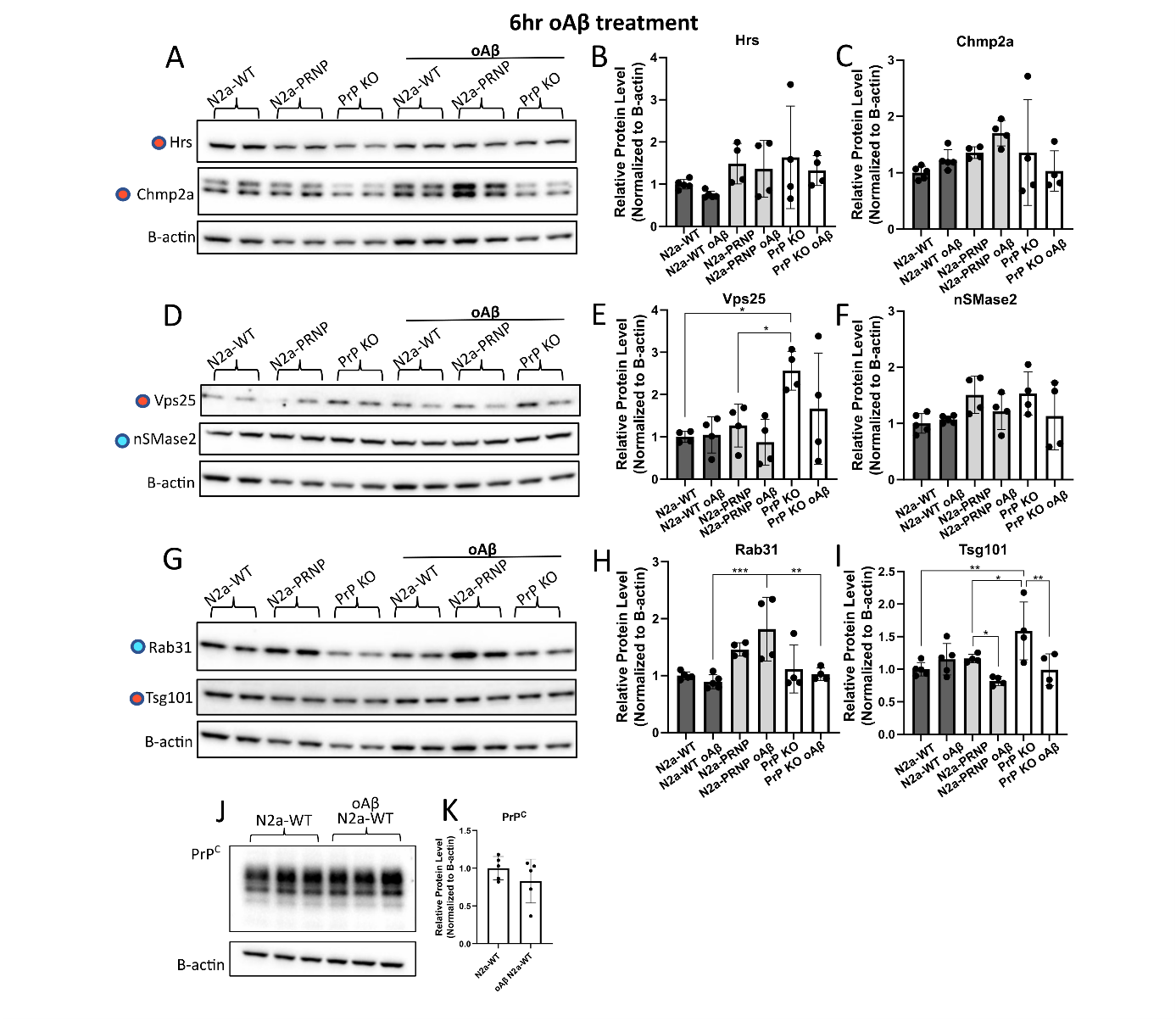


**Supplementary Figure 3. oAβ treatment has limited effect on exosome-biogenesis related proteins and PrP^C^ level after 6hrs.** Stimulating N2a-WT, N2a-PRNP and PrP KO cells for 6hrs with 2.5µM oAβ did not significantly affect the levels of most A-I) exosome-biogenesis related proteins or J,K) PrP^C^ level, demonstrated by western blot quantification. However, G,I) Tsg101 demonstrated a significant decrease upon oAβ stimulation. There were also significant differences between the different non-treated cell lines for D,E) Vps25 and G,H) Rab31 at this timepoint. ESCRT-dependent proteins are marked with a red dot and ESCRT-independent proteins are marked with a blue dot. *p<0.05, **p<0.01, ***p<0.001, two-way ANOVA with Fisher’s LSD test or Student’s t-test, n=4-5 independent biological replicates. Data presented as mean±SD.


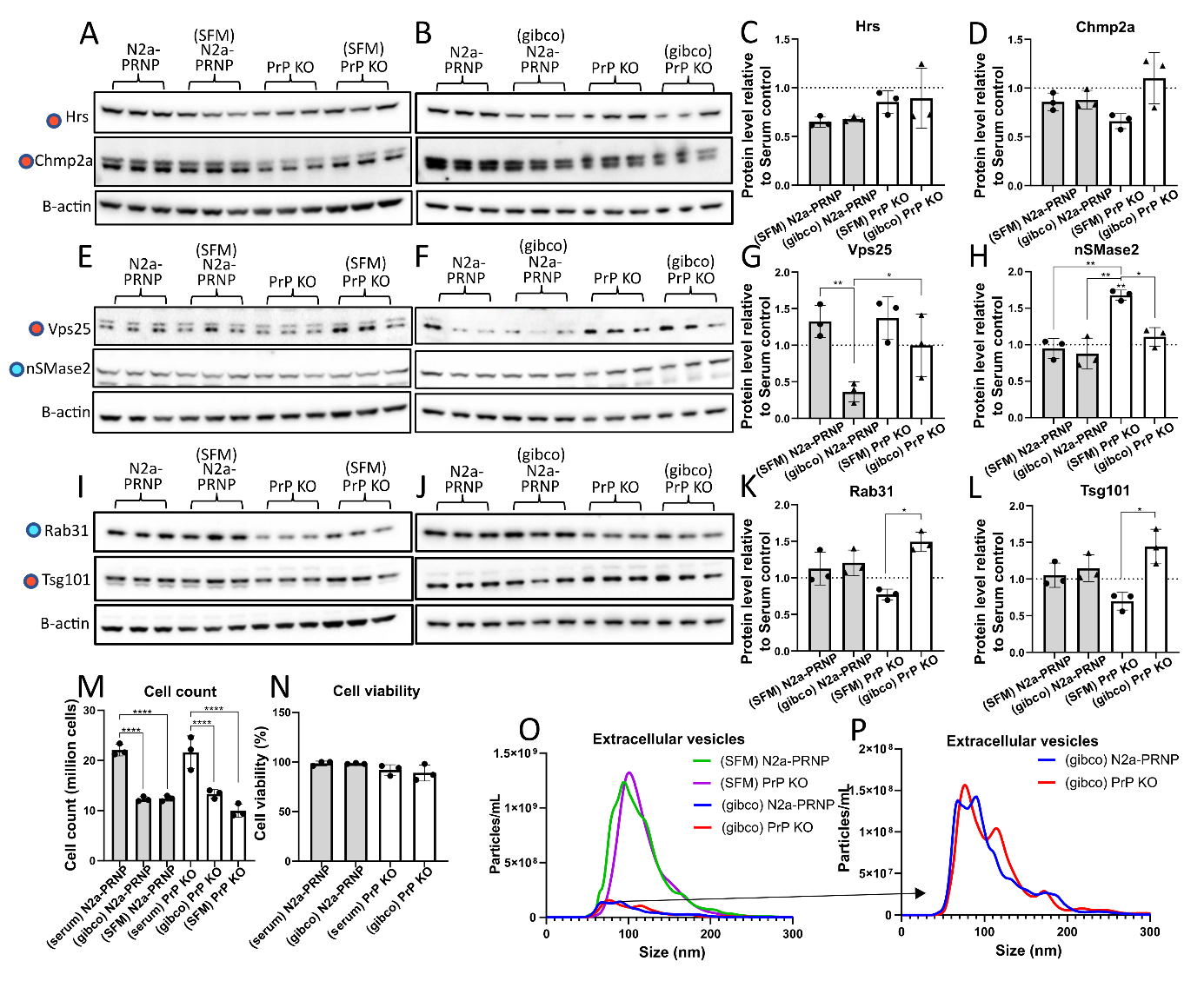


**Supplementary Figure 4. Serum free medium and EV-depleted serum medium affect exosome biogenesis-related proteins and EV abundance.** 48hr incubation with either serum free medium (SFM) or EV-depleted serum medium (gibco) was compared to regular serum medium in N2a-PRNP and PrP KO cells.A-L) Western blot quantification demonstrated several differences in levels of various exosome biogenesis- related protein levels when compared to regular serum medium (dotted line) and normalized to β-actin. M-N) Compared to regular serum medium, the serum free and EV-depleted serum medium led to decreased cell count which was not caused by a decrease in cell viability. O-P) EVs were also isolated from either serum free cell medium or EV-depleted serum cell medium by sequential ultracentrifugation showing significant differences in EV abundance. ESCRT-dependent proteins are marked with a red dot and ESCRT-independent proteins are marked with a blue dot. *p<0.05, **p<0.01, ****p<0.0001, two-way ANOVA with Fisher’s LSD test, n=3 independent biological replicates. Data presented as mean±SD.


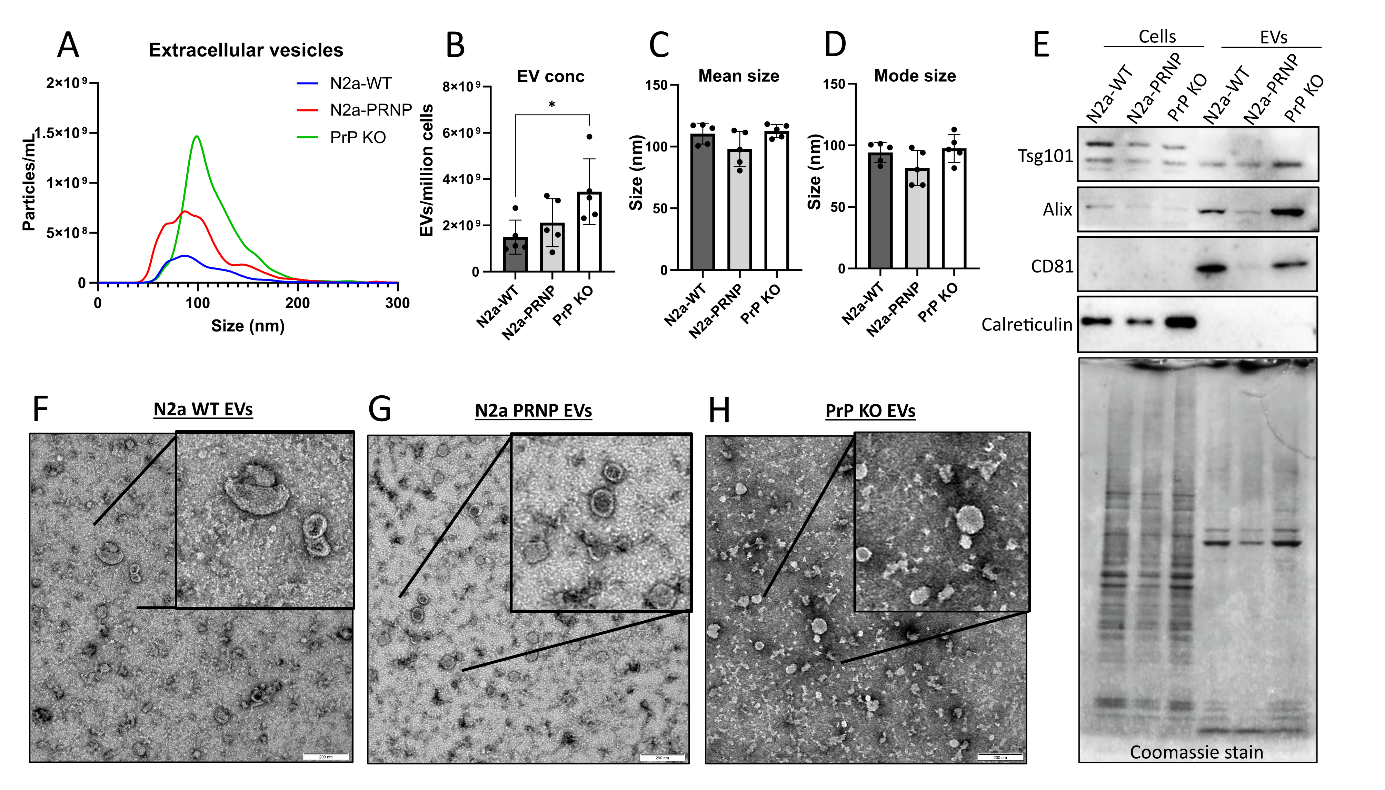


**Supplementary Figure 5. EV characterization.** Extracellular vesicles (EVs) were isolated from cell medium of N2a-WT, N2a-PRNP and PrP KO cells using sequential ultracentrifugation. A-B) NTA analysis revealed significant differences in concentration between PrP KO-derived and N2a-WT-derived EVs, B) but no significant difference in neither mean nor mode sizes. E) Western blot analysis demonstrated positive markers for Tsg101, Alix and CD81 in the EV samples. The EVs also had no cell contamination as demonstrated by the negative marker Calreticulin F-H) Electron microscopy images showed distinct EV morphology (highlighted by enlarged image sections), scale bar 200nm. *p<0.05, one-way ANOVA with Tukey post-hoc test, n=5 independent biological replicates. Data presented as mean±SD.


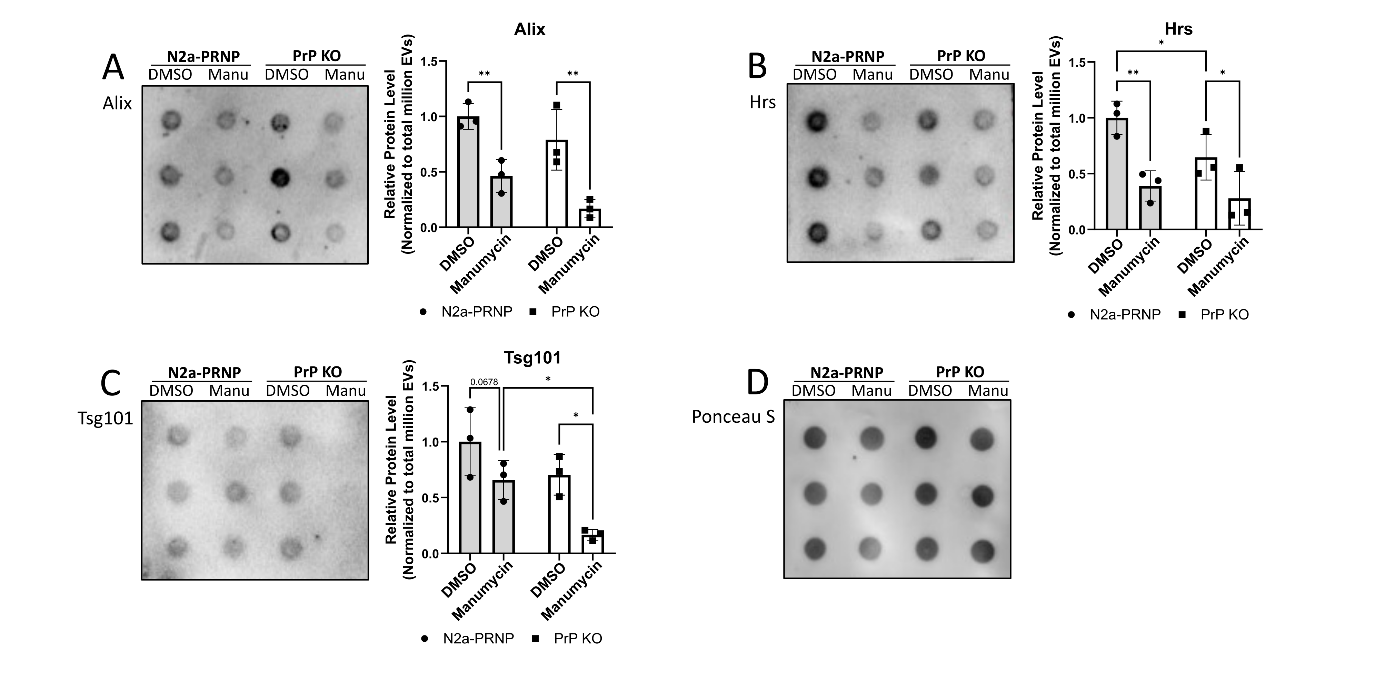


**Supplementary Figure 6. Manumycin A decrease ESCRT-related proteins in EVs.** A-C) Dot blot analysis demonstrates that 48hr exosome inhibition, using 0.25µM Manumycin A, resulted in a decreased level of Alix, Hrs and Tsg101, in extracellular vesicles (EVs) isolated from both N2a-PRNP and PrP KO cells. D) Ponceau S staining demonstrated equal loading of protein samples. *p<0.05, **p<0.01, two-way ANOVA with Fisher’s LSD test, n=3 independent biological replicates. Data presented as mean±SD.


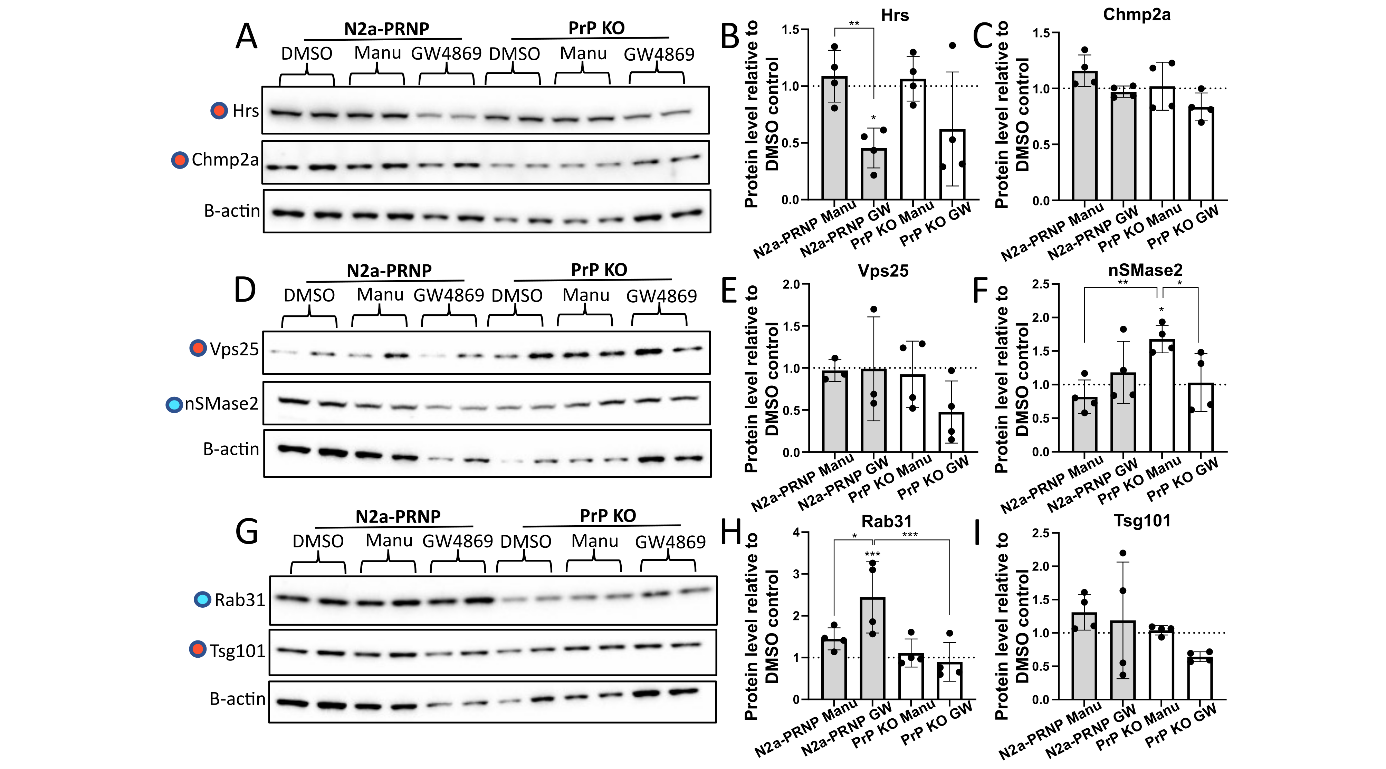


**Supplementary Figure 7. Effect of exosome-inhibitors on exosome-biogenesis related proteins.** N2a-PRNP and PrP KO cells were treated for 48hr with exosome inhibitors Manumycin A (Manu) and GW4869, or DMSO control. This resulted in differences in exosome biogenesis-related protein levels for A,B) Hrs, D,F) nSMase2, and G,H) Rab31. Manumycin A and GW4869-treated cells were related to their own DMSO cell model control (dotted line) and normalized to β-actin. ESCRT-dependent proteins are marked with a red dot and ESCRT-independent proteins are marked with a blue dot. * p<0.05, **p<0.01, ***p<0.001, two-way ANOVA with Fisher’s LSD test, n=4 independent biological replicates. Data presented as mean±SD.

**Supplementary References**

1. Akeus P, Szeponik L, Langenes V, Karlsson V, Sundström P, Bexe-Lindskog E, et al. (2021). Regulatory T cells reduce endothelial neutral sphingomyelinase 2 to prevent T-cell migration into tumors. Eur J Immunol. 51:2317–29.

2. Liang JW, Fang ZY, Huang Y, Liuyang ZY, Zhang XL, Wang JL, et al. (2018). Application of Weighted Gene Co-Expression Network Analysis to Explore the Key Genes in Alzheimer’s Disease. J Alzheimer’s Dis. 65:1353–64.

3. Kaczmarczyk L, Mende Y, Zevnik B, Jackson WS. (2016). Manipulating the prion protein gene sequence and expression levels with CRISPR/Cas9. PLoS One. 11:e0154604.

4. Torres M, Medinas DB, Matamala JM, Woehlbier U, Cornejo VH, Solda T, et al. (2015). The protein-disulfide isomerase ERp57 regulates the steady-state levels of the prion protein. J Biol Chem. 290:23631–45.
