# Supplementary material for: Lack of cellular prion protein causes Amyloid β accumulation, increased extracellular vesicle abundance, and changes to exosome biogenesis proteins
Source: Mol Cell Biochem. 2024 Jul 6;480(3):1569–82. doi: 10.1007/s11010-024-05059-0 (PMC11842432; doi:10.1007/s11010-024-05059-0)

Lack of cellular prion protein causes Amyloid β accumulation, increased extracellular vesicle abundance, and variations in exosome biogenesis proteins

Lovisa Johansson[1*], Juan F. Reyes[1], Tahir Ali[2,3], Hermann Schätzl[2,3], Sabine Gilch[2,3], Martin Hallbeck[1*]

[1]Department of Biomedical and Clinical Sciences and Department of Clinical Pathology, Linköping University, Linköping, Sweden.

[2] Calgary Prion Research Unit, Faculty of Veterinary Medicine, University of Calgary, Calgary, Alberta, Canada.

[3] Hotchkiss Brain Institute, University of Calgary, Calgary, Alberta, Canada.

*Corresponding authors

Corresponding authors' emails: Lovisa.Johansson@liu.se, Martin.Hallbeck@liu.se

Original western blot images for main figures

Figure 1A

Hrs

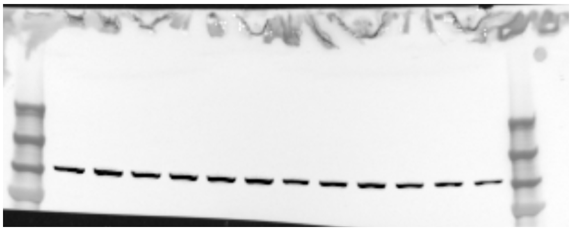

Chmp2a

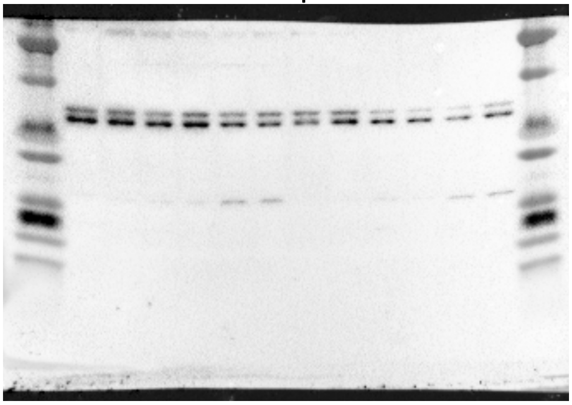

β-Actin

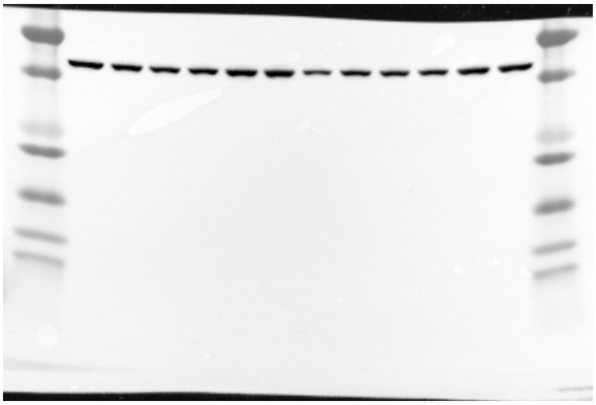

Figure 1D

Vps25

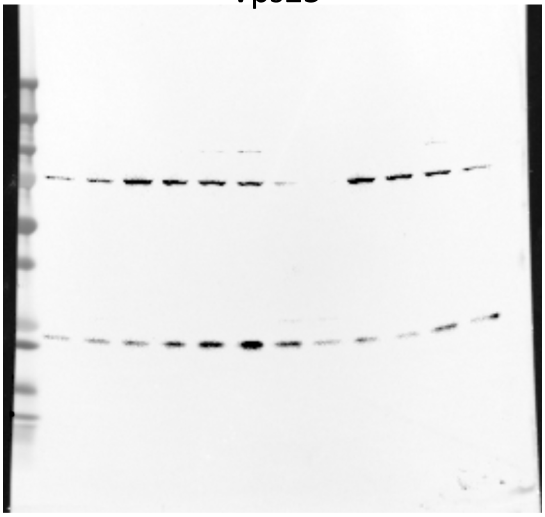

β-Actin

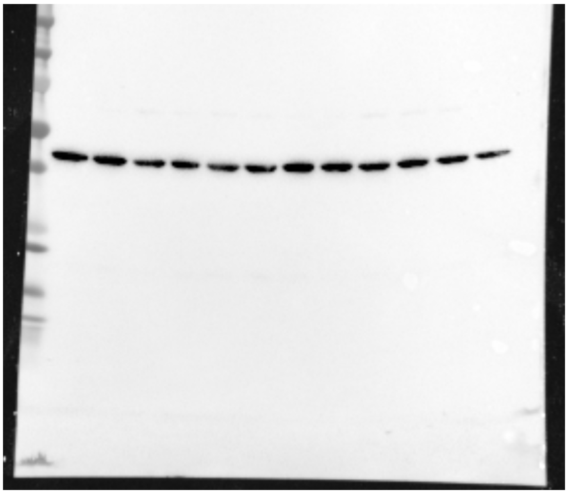

nSmase2

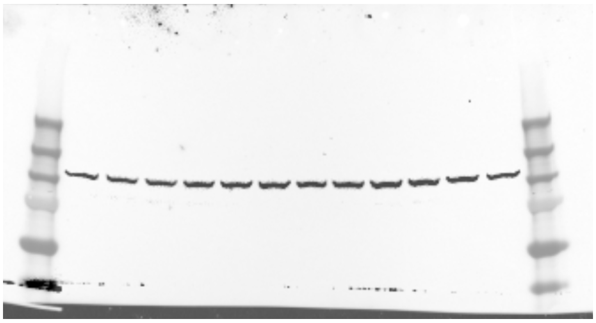

β-Actin

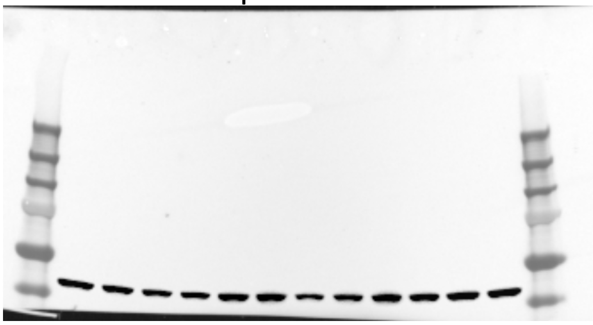

Original western blot images for main figures

Figure 1G

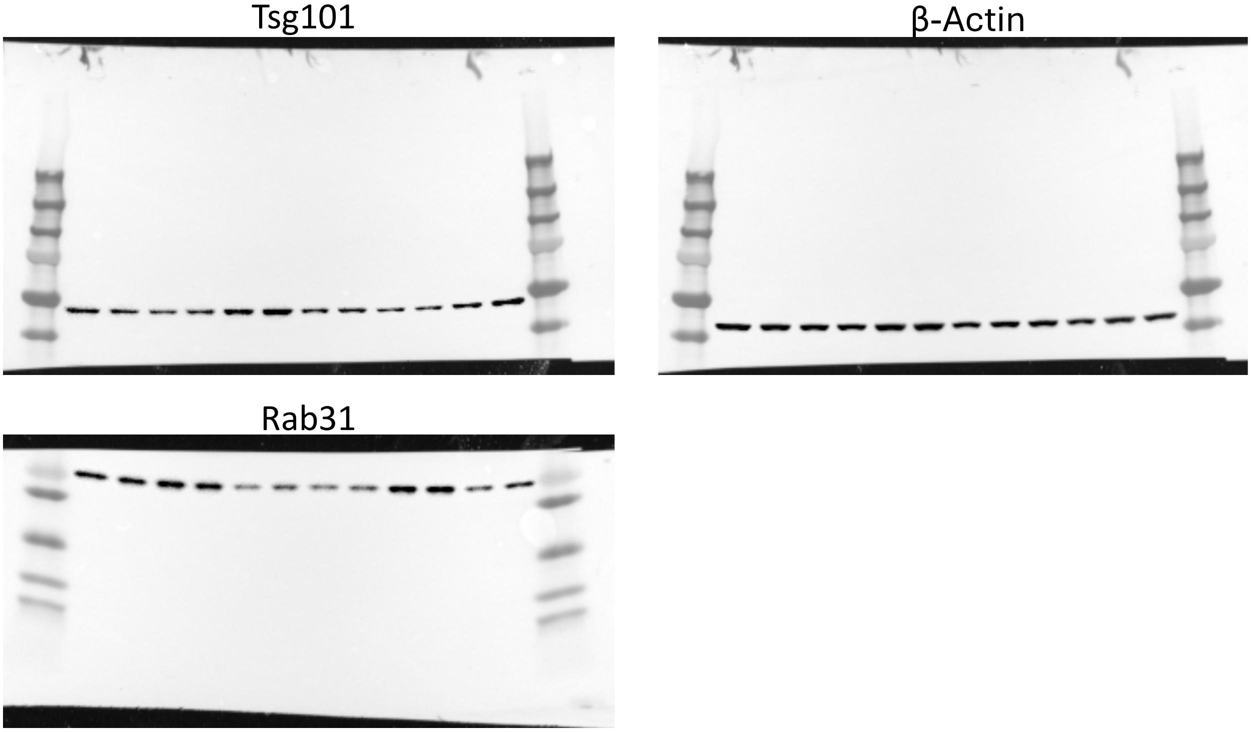

Figure 1J

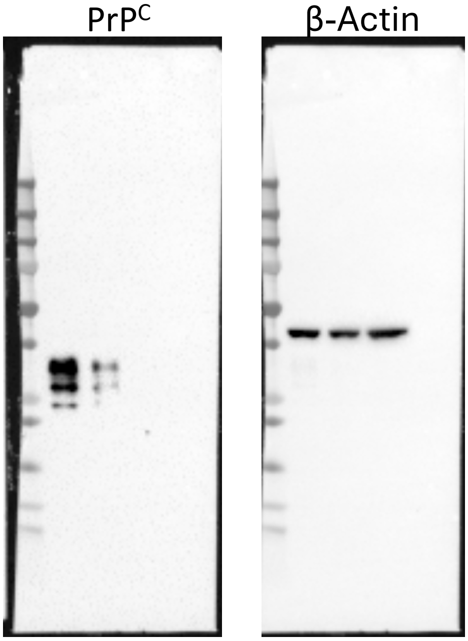

Figure 1L

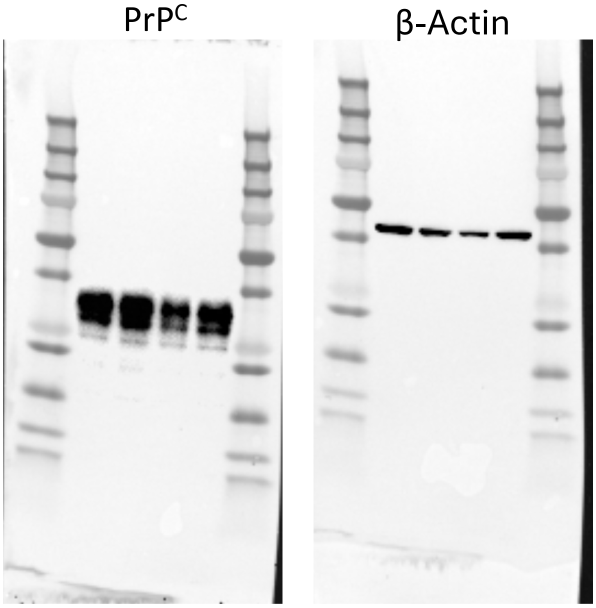

Original western blot images for main figures

Figure 4A

PrP^C

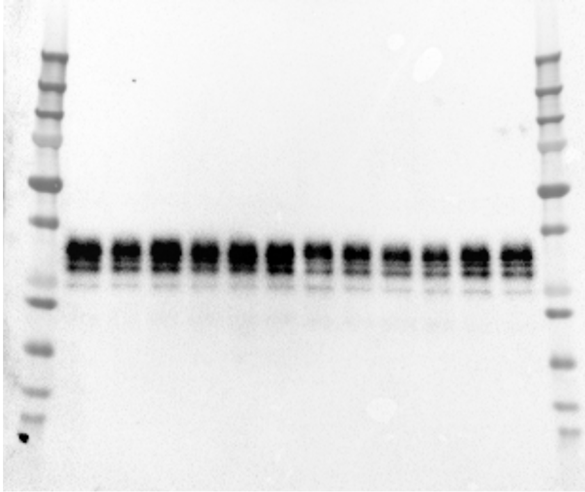

β-Actin

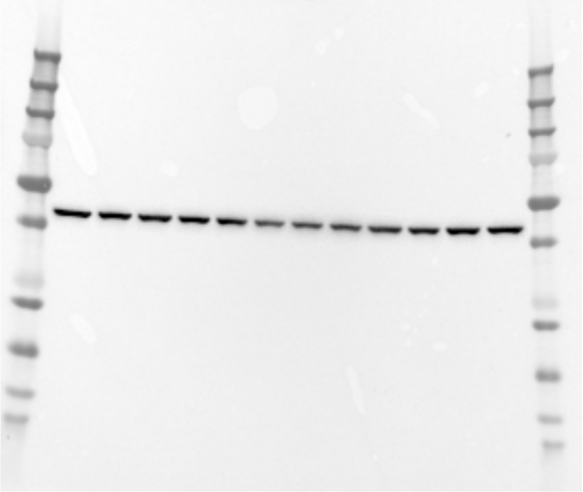

Figure 4B

PrP^C

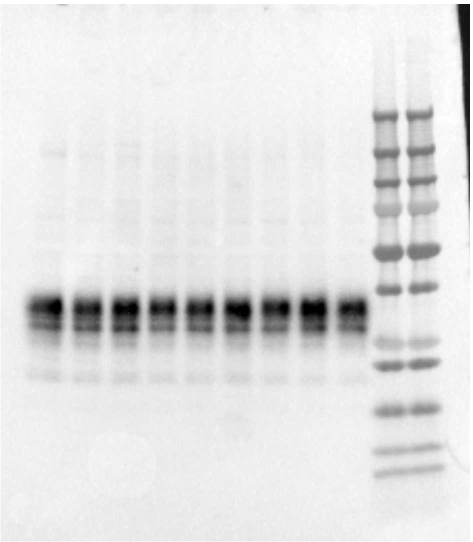

β-Actin

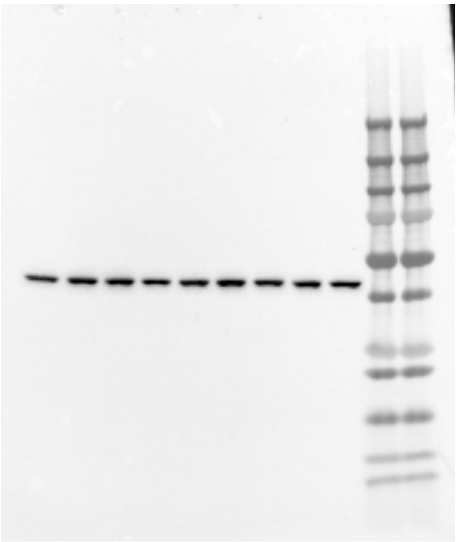

Original western blot images for main figures

Figure 4C

Hrs

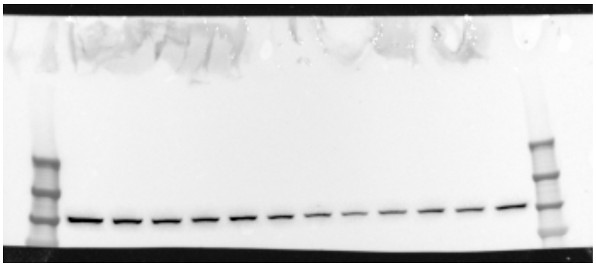

Chmp2a

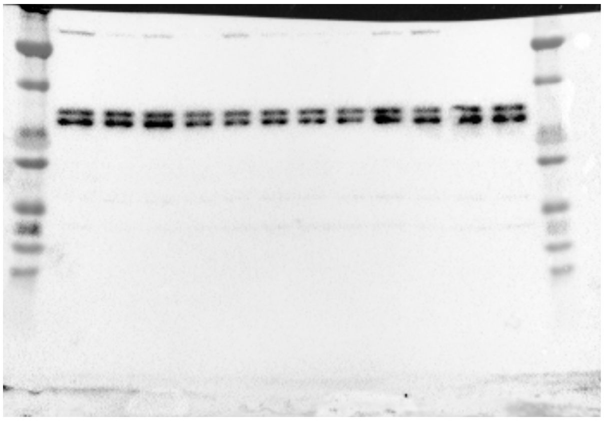

β-Actin

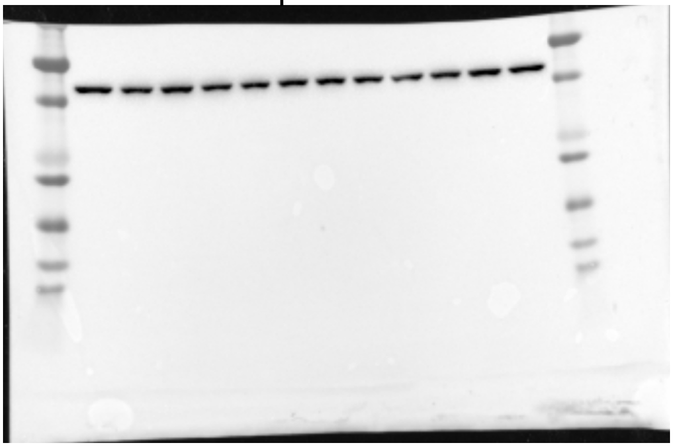

Figure 4D

nSmase2

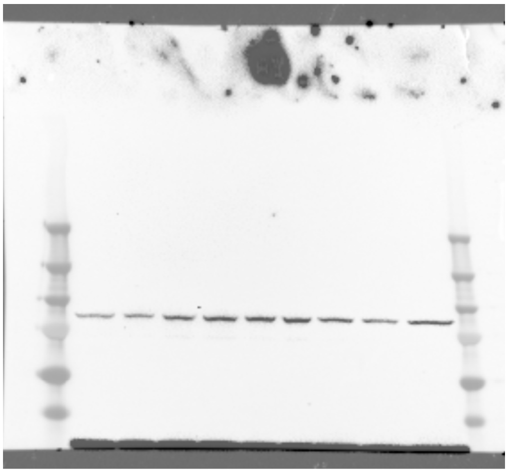

β-Actin

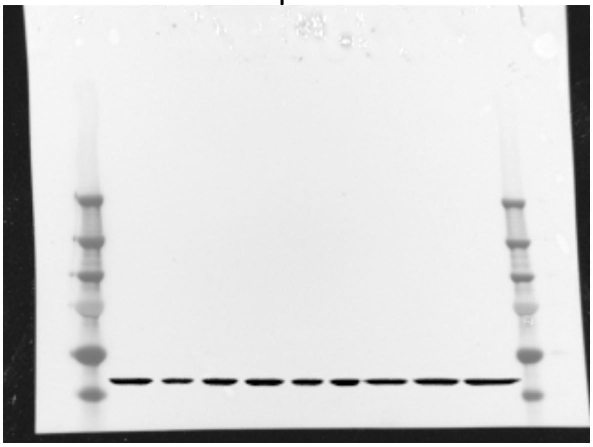

Vps25

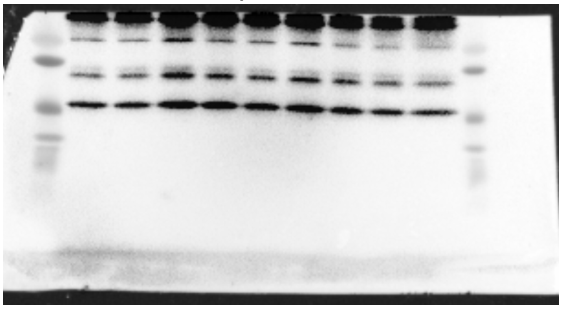

Original western blot images for main figures

Figure 4E

Tsg101

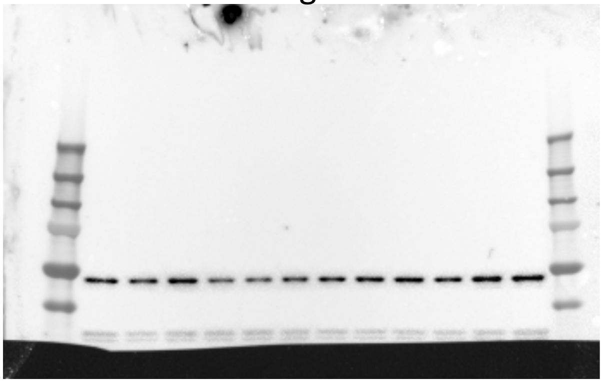

β-Actin

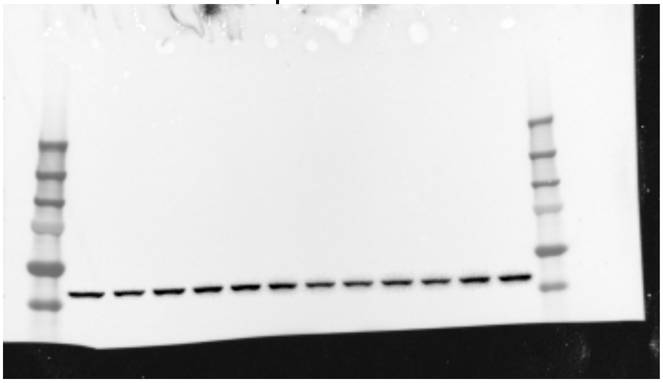

Rab31

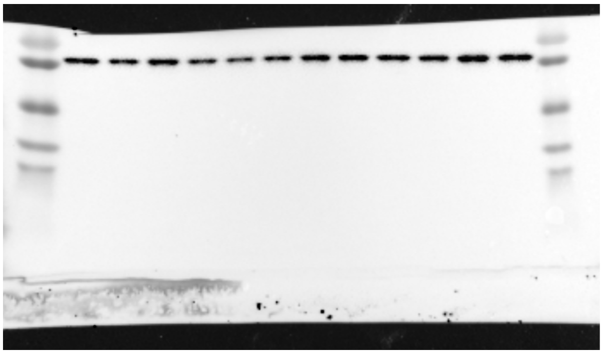

Supplement: Supplementary file 2 — Supplementary file2 (PDF 4486 kb) [file 11010_2024_5059_MOESM2_ESM.pdf]
